# The Development of Carbon/Silicon Heterojunction Solar Cells through Interface Passivation

**DOI:** 10.1002/advs.202306993

**Published:** 2024-01-17

**Authors:** Bingbing Chen, Xuning Zhang, Qing Gao, Dehua Yang, Jingwei Chen, Xuan Chang, Cuili Zhang, Yuhua Bai, Mengnan Cui, Shufang Wang, Han Li, Benjamin S. Flavel, Jianhui Chen

**Affiliations:** ^1^ Advanced Passivation Technology Lab College of Physics Science and Technology Hebei University Baoding 071002 China; ^2^ Province‐Ministry Co‐Construction Collaborative Innovation Center of Hebei Photovoltaic Technology College of Physics Science and Technology Hebei University Baoding 071002 China; ^3^ Institute of Nanotechnology Karlsruhe Institute of Technology Kaiserstrasse 12 76131 Karlsruhe Germany

**Keywords:** carbon/silicon heterojunction solar cells, conductive passivating contact, high efficiency, interface passivation, low cost

## Abstract

Passivating contactsin heterojunction (HJ) solar cells have shown great potential in reducing recombination losses, and thereby achieving high power conversion efficiencies in photovoltaic devices. In this direction, carbon nanomaterials have emerged as a promising option for carbon/silicon (C/Si) HJsolar cells due to their tunable band structure, wide spectral absorption, high carrier mobility, and properties such as multiple exciton generation. However, the current limitations in efficiency and active area have hindered the industrialization of these devices. In this review, they examine the progress made in overcoming these constraints and discuss the prospect of achieving high power conversion efficiency (PCE) C/Si HJ devices. A C/Si HJ solar cell is also designed by introducing an innovative interface passivation strategy to further boost the PCE and accelerate the large area preparationof C/Si devices. The physical principle, device design scheme, and performanceoptimization approaches of this passivated C/Si HJ cells are discussed. Additionally, they outline potential future pathways and directions for C/Si HJ devices, including a reduction in their cost to manufacture and their incorporation intotandem solar cells. As such, this review aims to facilitate a deeperunderstanding of C/Si HJ solar cells and provide guidance for their further development.

## Graphical abstract

In this review, the development of carbon/silicon heterojunction (C/Si HJ) solar cells is examined through interface passivation and discuss the prospect of those devices, aiming to facilitate a deeper understanding of C/Si HJ solar cells as well as providing guidance for their further development.

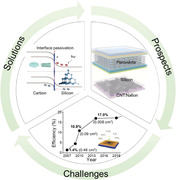

## 1 Background

Solar energy is considered one of the most promising renewable energy sources to reduce the consumption of fossil fuels and achieve carbon neutrality. Solar cell devices, including crystalline silicon (c‐Si) solar cells,^[^ ^1^, ^2^ ^]^ copper indium gallium selenium (CIGS),^[^ ^3^ ^]^ cadmium telluride (CdTe),^[^ ^4^ ^]^ organic solar cells^[^ ^5^ ^]^ and perovskite solar cells,^[^ ^6^ ^]^ have advanced rapidly and are striving to meet the increasing demand for clean energy. Owing to their high power conversion efficiency (PCE), long stability, and scalable mass production techniques, Si solar cells occupy more than 95% of the worldwide photovoltaic (PV) market.^[^ ^7^, ^8^ ^]^

**Figure 1:**
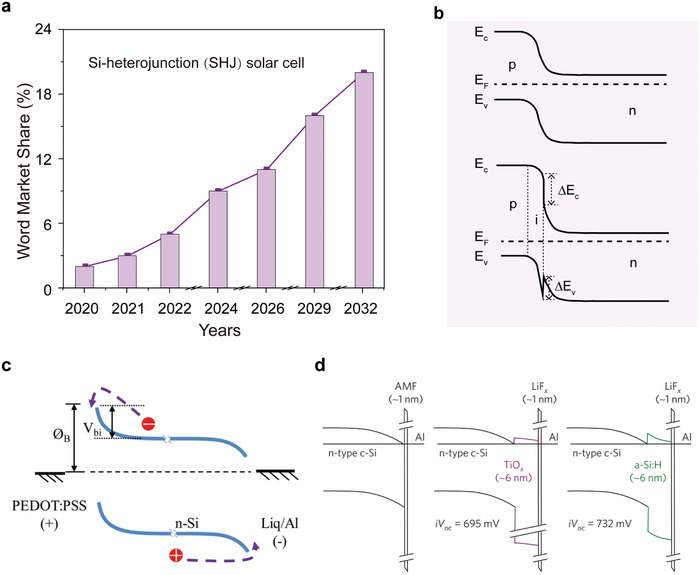
The characteristic of current c‐Si solar cell technology. a) The photovoltaic market share of c‐Si solar cells.^[^ ^24^ ^]^ b) The energy level schematic diagram of Si‐Si homojunction (top) and HJ (bottom), E_c_, E_v_, E_f_, ΔE_c_, and ΔE_v_ denote the conduction band, the valence band, the fermi level, the conduction band offset, and the valence band offset, respectively. The energy level schematic diagram of c) A PEDOT:PSS/Si solar cell^[^ ^35^ ^]^ and d) a dash solar cell,^[^ ^38^ ^]^ respectively. Copyright 2015, Elsevier for Energy Procedure. Copyright 2016, IEEE for james bullock.

The PV effect at a p‐n junction is at the heart of c‐Si solar cells. A p‐n homojunction is formed by diffusing a p (or n) emitter onto a n (or p) Si substrate. The aluminum back surface field (Al‐BSF) cell, in which the entire rear silicon surface is alloyed with aluminum to form the device's positive terminal, is one such solar cell that has dominated large‐scale industrial PV device production for the last 30 years. This is due to its simple design and low‐cost to manufacture.^[^ ^9^, ^10^, ^11^ ^]^ However, recombination losses and a high series resistance caused by metal‐semiconductor interfaces result in a low open‐circuit voltage (*V*_OC_) and a low fill factor (FF), which combined limit the PCE of Al‐BSF cells to only ≈20%.^[^ ^12^ ^]^ A passivated emitter and rear cell (PERC) design was subsequently proposed and this has enhanced the PCE of Si solar cells up to 25%.^[^ ^8^, ^13^, ^14^, ^15^, ^16^, ^17^ ^]^ However, the metal–silicon contact still exists in PERC and thus the cells still suffer from recombination losses of the photogenerated electrons and holes.^[^ ^18^ ^]^ To mitigate the problems associate with metal‐semiconductor contacts, the so‐called passivating contact was proposed. This involves the insertion of ultrathin passivation films, normally silicon oxide (SiO_x_) or hydrogenated amorphous silicon (a‐Si:H), between the Si wafer and the metal electrode.^[^ ^19^, ^20^ ^]^ Owing to these passivating contact techniques, silicon HJ (SHJ) and tunnel oxide passivated contact (TOPCon) cells are considered the most promising next‐generation PV cell technologies^[^ ^21^, ^22^ ^]^ and, LONGi recently reported a new record PCE of 26.81% on SHJ cells.^[^ ^23^ ^]^ Moreover, the global market share for SHJ solar cells has steadily grown (**Figure** **1**a),^[^ ^24^ ^]^ and SHJ solar cells are expected to have a market share of ≈10% after 2024 and close to 20% by 2032.

The energy level diagram of both p‐n homojunction and HJ solar cells is shown in Figure 1b. In both cases photons are absorbed by the Si wafer, these create photogenerated electrons and holes which are separated and extracted by carrier‐selective transport layers. For the p‐n homojunction, a high temperature (> 800 °C) p (or n) type doped silicon region acts as the carrier selective transport layer.^[^ ^25^ ^]^ SHJ solar cells use an thin intrinsic a‐Si:H(i) layer as a ultrathin passivation interlayer, and a doped a‐Si:H (n^+^ or p^+^) as a carrier selective contact layer. The carrier selective contact is formed without high‐temperature process and this is a potential benefit for the mainstream c‐Si PV industry,^[^ ^26^ ^]^ however, SHJ devices still require expensive vacuum equipment to prepare the highly doped (n+ or p+) silicon regions.^[^ ^27^ ^]^ For the next generation of solar cells, the structural complexity and ease of device fabrication will be the key factors in determining the cost and viability of Si‐based devices.

The use of alternative materials which are dopant free such as organic films (e.g., PEDOT:PSS),^[^ ^28^, ^29^ ^]^ transition metal oxides (e.g., MoO_x_),^[^ ^30^, ^31^ ^]^ metallic salts (e.g., LiF) and sulphides (e.g., ZnS)^[^ ^32^ ^]^ for the development of HJs devices are a particularly attractive pathway for solar cell manufacture. In 2014, 8‐hydroxyquinolinolato‐lithium (Liq) was introduced at the rear side of Si substrate to reduce charge recombination and the cells achieved a high PCE of 12.2%.^[^ ^33^ ^]^ In 2016, He et al. presented a fully conformal contact by a post‐fabrication coating of diethyl phthalate (DEP) and were able to demonstrate efficiencies above 16%.^[^ ^34^ ^]^ Figure 1c shows the energy band diagram of PEDOT:PSS/Si HJ.^[^ ^35^ ^]^ Record‐high efficiencies of 18.3% and 20.6% of PEDOT:PSS/Si HJ solar cells were achieved by Zielke et al. on n‐type silicon and p‐type silicon wafers,^[^ ^35^ ^]^ respectively. On the other hand, Bullock et al. developed and implemented dopant‐free asymmetric hetero contacts (DASH cells) using alkali metal fluorides and metal oxides and these resulted in PCEs approaching 20% (Figure 1d).^[^ ^35^ ^]^ Yang et al. reported that a thin film of TiO_x_N_y_ which simultaneously provides moderate surface passivation and enabled a low contact resistivity on c‐Si surfaces. In their work, a PCE of 22.3% was achieved for a c‐Si solar cell featuring a full‐area dopant‐free electron‐selective contact.^[^ ^36^ ^]^ Currently the highest conversion efficiency achieved by DASH cells is 23.5% and this places them into a realm that is competitive with c‐Si cell architectures.^[^ ^37^ ^]^ Unfortunately, the mainstream passivating thin films used in common DASH cells, such as a‐Si:H, still require the use of expensive equipment, and the complexity of the HJ device fabrication process is thus not significantly reduced.

## 2 Carbon/Silicon HJ Solar Cells

**Figure 2:**
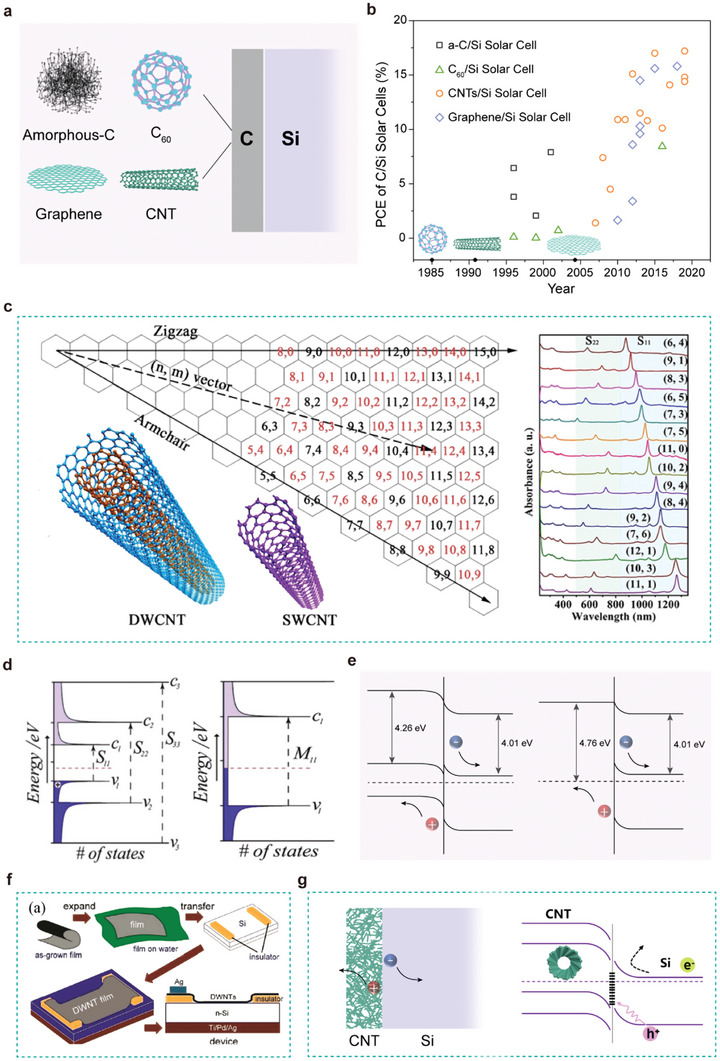
The characteristics of C/Si HJ solar cells. a) Four classes of carbon allotropes (a‐C, fullerene (C_60_)), CNTs, and graphene) formation of C/Si HJ solar cells.^[^ ^40^ ^]^ Copyright 2015, John Wiley and Sons for Advanced Materials. b) The timeline exhibits the evolution and selected major events of C/Si HJ and solar cells up to 2019. c) Chiral map of SWCNTs. Zigzag and armchair nanotubes correspond to SWCNTs with m = 0 and n = m, respectively. Optical absorption spectra of different semiconducting SWCNTs achieved by sorting are presented on the right. S_11_ and S_22_ represent the first and second optical transitions of SWCNTs with the diameter ranging from 0.8‐1.0 nm, respectively.^[^ ^56^ ^]^ Copyright 2020, John Wiley and Sons for Advanced Energy Materials. d) Schematic illustration of the DOS of s‐SWCNTs (left) and m‐SWCNTs (right) with the optical transitions between VHS.^[^ ^51^ ^]^ Copyright 2018, John Wiley and Sons for Advanced Energy Materials. e) The energy‐band diagrams of the CNT/Si HJ solar cells based on p‐n junction or Schottky junction solar cells.^[^ ^57^ ^]^ Copyright 2012, John Wiley and Sons for Advanced Energy Materials. f) Illustration of the fabrication process of DWNT/n‐Si HJ solar cell.^[^ ^77^ ^]^ Copyright 2012, John Wiley and Sons for Advanced Energy Materials. g) The energy level diagram of C/Si HJ solar cells.

Similar to Si, carbon (C) is an abundant element in nature, and the exploration of carbon materials is closely connected to human history. Owing to their excellent carrier mobility, chemical stability, and optoelectronic properties, carbon materials fulfill all of the requirements for solar cell manufacture in combination with silicon.^[^ ^39^, ^40^ ^]^ Generally, a C/Si HJ solar cell consists of a carbon layer (carrier‐selective contact layer) and a Si (light absorbing layer) as well as front and rear metal electrodes. At present, the most common carbon films used in C/Si HJ solar cells are amorphous carbon (a‐C), graphite, graphene, fullerene, and carbon nanotubes (CNTs) as shown in **Figure** **2**a.^[^ ^40^ ^]^ The development of the C/Si HJ solar cells was initially slow due to the technical difficulties to integrate carbon materials. As such and until recently, the C/Si HJ device geometry was highly unusual when compared to commercial Si solar cells. In most cases, researchers employed an architecture resembling that of an organic solar cell with a window or frame‐like geometry defined in the middle of a Si wafer.^[^ ^41^ ^]^ In this geometry, a SiO_2_/Si wafer was etched to reveal a small silicon opening in the SiO_2_ and the surrounding SiO_2_ was coated with metal electrode (such as Au, Ag, or Pt/Ti). The use of a window or frame like geometry allowed for the carbon film to be processed separately and later transferred to the window and this design was successful for many years. Figure 2b and **Table** **1** displays the representative results of window‐like geometry C/Si solar cells from the year of invention to the year of 2019.

**Table 1:** The results of window‐like geometry C/Si HJ solar cells efficiency from the year of invention to 2019.

Solar cell structure	PCE [%]	Active area [cm^2^]	*V*_oc_ [mV]	*J*_sc_ [mA cm^−2^]	FF [%]	Year	First Institution
a‐C/Si	–	–	280	–	–	1979	Tsinghua University^[^ ^42^ ^]^
	3.8	0.28	325	2.73	65	1996	Tokai University^[^ ^43^ ^]^
	6.45	0.28	420	3.71	63	1996	Tokai University^[^ ^63^ ^]^
	2.07	0.25	479	18.4	31.8	1999	Nagoya Institute of Technology ^[^ ^39^ ^]^
	7.9	2	580	32.5	42	2001	Xinjiang University^[^ ^64^ ^]^
C_60_/Si	<0.1	–	400	–	30	1996	University of Tokyo^[^ ^65^ ^]^
	0.023	0.25	170	0.33	41.5	1999	Toyota Technological Institute^[^ ^66^ ^]^
	0.7	2.6	306	11.58	19.9	2002	Toyota Technological Institute^[^ ^67^ ^]^
	8.43	0.13	500	25.1	67	2016	Ulsan National Institute of Science and Technology^[^ ^68^ ^]^
Graphene/Si	1.65	0.1	480	6.5	56	2010	Tsinghua University^[^ ^69^ ^]^
	3.4	0.1	570	21.0	28	2012	Tsinghua University^[^ ^70^ ^]^
	8.6	0.09	540	25.3	51	2012	University of Florida^[^ ^71^ ^]^
	9.6	0.1	550	16.91	72	2013	Tsinghua University^[^ ^72^ ^]^
	10.3	0.04	480	38.86	55	2013	Hefei University of Technology^[^ ^73^ ^]^
	14.5	0.047	612	32.7	72	2013	Tsinghua University^[^ ^74^ ^]^
	15.6	0.11	595	36.7	72	2015	Tsinghua University^[^ ^75^ ^]^
	15.8	0.03	590	36.8	73	2018	Tianjin University^[^ ^76^ ^]^
CNT/Si	1.4	0.49	500	13.8	19	2007	Tsinghua University^[^ ^77^ ^]^
	7.4	0.49	540	26	53	2008	Tsinghua University^[^ ^78^ ^]^
	4.5	0.25	350	26.5	49	2009	Arkansas University^[^ ^79^ ^]^
	10.9	0.08	550	25	79	2010	Florida University^[^ ^80^ ^]^
	10.9	0.09	560	29	67.6	2011	Tsinghua University^[^ ^81^ ^]^
	15.1	0.15	610	32	77	2012	Peking University^[^ ^82^ ^]^
	11.5	0.09	530	29.3	74	2013	Yale University^[^ ^83^ ^]^
	10.8	0.49	510	31	69	2014	Yale University^[^ ^84^ ^]^
	17	0.00785	590	36.6	78	2015	Kyoto University^[^ ^85^ ^]^
	10.11	2.15	630	25.32	51.97	2016	Peking University^[^ ^86^ ^]^
	14.09	0.09	540	36.1	72.3	2017	Kyoto University^[^ ^87^ ^]^
	14.8	0.09	618	33.7	71.2	2019	Peking University^[^ ^88^ ^]^
	14.4	0.09	549	36.7	71.2	2019	Kyoto University^[^ ^89^ ^]^
	17.2	1	659	32.3	76.3	2019	Karlsruhe Institute of Technology^[^ ^90^ ^]^

Note: The illumination intensity of ref. [35] is 15 [mW cm^−2^] (xenon arc lamp).

The first C/Si HJ PV effect was proposed and reported by Bhagavat and Nayak in 1979.^[^ ^42^ ^]^ A HJ was prepared by depositing an amorphous carbon (a‐C) film onto an n‐Si substrate. This HJ showed a rectification ratio of two orders of magnitude and put out a photovoltage of 280 mV. In 1996, Yu et al. reported a more complex a‐C/Si HJ solar cell; including a back electrode, n‐type Si substrate, a‐C film, and Au electrode and achieved 2.73 mA cm^−2^ of short‐circuit current, 325 mV of *V*_OC_, FF of 65%, and PCE of 3.8%.^[^ ^43^ ^]^ It was this work which then initiated the study of the C/n‐Si layer for photovoltaics. Over the following 20 years, efforts were then devoted to enhancing the PCE of the a‐C/Si HJ devices. However, difficulties in controlling the sp^2^/sp^3^ ratio of the a‐C and the inhomogeneity of dopants as well as the extreme growth process of a‐C, have hindered the device performance improvement. Currently, the record PCE of the a‐C/Si HJ solar cell is 7.9% with an active area of 2 cm^2^.

The use of nanomaterials offers a solution and work combining zero‐dimensional fullerene (C_60_) in a (C_60_)/Si HJ has shown good rectifying properties, and by doping the C_60_ layer, surface passivation and the incorporation of anti‐reflection coatings, a PCE of 8.43% (active area of 0.13 cm^2^) was achieved at a C_60_/p‐Si solar cell by Yun et al. in 2016.^[^ ^44^ ^]^ However, the limited intrinsic conductivity of C_60_, makes the further development of the C_60_/Si solar cell challenging.

Unlike a thin film of C_60_ with a high resistance, graphene has an electron mobility of 2.5 × 10^5^ cm^2^ V^−1^ S^−1^,^[^ ^45^ ^]^ which is hundreds of times higher than that of silicon. In 2010, Li et al. reported the development of a two‐dimensional carbon atomic layer graphene/Si solar cell.^[^ ^46^ ^]^ Building upon previous research on other C/Si HJ solar cells, the development of graphene/Si solar cells progressed quickly and Ma et al. reported a PCE of 15.8% in a graphene/MoS_2_/Si Schottky barrier solar cell.^[^ ^46^, ^47^ ^]^

Among the carbon allotropes, CNTs with their unique structure and electrical properties (a metallic (m) or semiconducting (s) property) boast a host of properties that also make them attractive for PV. Conceptually, CNTs are 1D cylindrical molecules with a diameter of a few nanometers. A single‐wall carbon nanotube (SWCNT) can be theoretically considered as a rolled‐up form of graphene along a specific lattice vector (*n, m*). Double‐wall carbon nanotubes (DWCNTs) and multi‐wall carbon nanotubes (MWCNTs) consist of two or more coaxially SWCNTs. A series of (*n, m*) indices defining the atomic structure of CNTs are shown in Figure 2c. The integers (*n, m*) originate from the chiral vector, C_h_ = *n*a_1_ + *m*a_2_, which describes the number of steps along the graphene lattice basis vectors (a_1_ and a_2_) in real space.^[^ ^48^ ^]^ Slight differences in structure can lead to significant differences in the properties of various SWCNTs. For example, electronic band structure calculations predict that the (*n, m*) indices determine the metallic or semiconducting behavior of SWCNTs.^[^ ^49^, ^50^ ^]^ Zigzag (*n, 0*) SWCNTs should have two distinct types of behavior: the tubes will be metals when n/3 is an integer, and otherwise semiconductors. As C_h_ rotates away from (*n, 0*), chiral (*n, m*) SWCNTs are possible with electronic properties similar to the zigzag tubes; that is, when (2n+m)/3 is an integer the tubes are metallic, and otherwise semiconducting. The typical density of electronic states (DOS) of metallic SWCNTs (m‐SWCNTs) and semiconducting SWCNTs (s‐SWCNTs) are exhibited in Figure 2d.^[^ ^51^ ^]^ Unlike bulk materials, maximum points of DOS, namely the van Hove singularities, arise in 1D nanotubes and, as a first approximation, play a significant role in the optical properties and electrical transport of SWCNTs. As shown in Figure 2c, distinct sharp optical adsorption peaks corresponding to the optical transitions between symmetrical van Hove singularities are observed due to the discrete distributions of electronic states. The band gap of a SWCNT varies inversely with its diameter, and the wavelength of the optical adsorption peaks of s‐SWCNTs increases with diameter, extending into the visible and near‐infrared bands beyond that of silicon. Among the various potential SWCNT applications is photovoltaics and the SWCNT/Si HJ is one of the simplest and most scalable approaches. The work function of a SWCNT varies with the different diameter and can range from ≈4.5 to 5.1 eV,^[^ ^52^ ^]^ and form a hole‐selective contact with n‐Si. Therefore, by coating a Si substrate with a SWCNT film, a typical SWCNT/Si HJ can be fabricated at room temperature. Additionally, the intrinsic mobility of semiconducting SWCNTs, which is as high as 105 cm^2^ V s^−1^,^[^ ^53^, ^54^ ^]^ makes SWCNT films a potential high‐conductive path for carriers. Moreover, the photocurrent excited by near‐infrared light in SWCNT devices suggests that SWCNT film may serve as photo‐sensing materials as well as carriers collecting and transporting layers in SWCNT/Si solar cells.^[^ ^55^ ^]^ Up to now, the window‐like geometry CNT/Si solar cells have been developed from 1.4% PCE in first reports to 17.2% with 1 cm^2^ device areas,^[^ ^56^ ^]^ which is significantly higher than that of other carbon‐based Si devices. However, as mentioned above, raw SWCNT materials consist of various (*n, m*) species with different properties, leading to the complex work mechanism of HJs in an SWCNT device.

CNT materials will respectively form two types of HJs when in contact with Si wafers: a p–n (semiconductor/semiconductor) junction or Schottky (metal/semiconductor) junctions. The energy band diagrams of the CNT/Si HJ solar cells with p‐n junction or Schottky junction are displayed in Figure 2e.^[^ ^57^ ^]^ When a CNT/Si HJ is illuminated, photons with energy greater than the bandgap can excite electrons in the valence band to the conduction band, creating holes in the valence band where the excited electrons are previously located and then photogenerated carriers can be separated under the built‐in field. Positive charges will accumulate near the surface of the silicon side and negative charges transfer to the other side near the p‐C layer. When the density of interface states is low enough for both the p‐n junction and Schottky junctions, the built‐in fields can be critically adjusted by the work function of the carbon layer. If the work function of the carbon increases, a higher built‐in potential will be formed, enhancing the junction's capacity to collect photogenerated carriers. Unlike traditional PERC or SHJ solar devices, carbon films can be prepared by solution‐process technologies at room temperature and in the atmospheric environment,^[^ ^58^ ^]^ making it possible to mass‐produce CNT/Si HJ solar cells in large quantities by a simple and low‐cost solution process.^[^ ^58^ ^]^

Although many technologies with electrical and optical design have been used to improve the CNT/Si HJ, two main limitations have hindered industrialization: the low PCE (<18%) and the small active area (<0.1 cm^2^) (as shown in Table 1). As shown in Figure 2f, the current method for preparing CNT/Si device with a window‐like geometry involves conformally transferring CNT films to a Si wafer. The area of this architecture is usually small (0.008–2 cm^2^), and the design is difficult to scale up without compromising PCE. Furthermore, there are many defect states at the CNT/Si interface (Figure 2g), where recombination of the photoinduced carriers can lead to poor performance and interface passivation is key to achieving a high PCE. Currently, classic passivation (chemical passivation and field‐effect passivation) techniques have been applied for high‐efficiency Si solar cells. An alternative passivation strategy is chemical passivation (passivation materials including SiO_2_, a‐Si:H) and is based on a covalent bond formed between the Si surface atoms and atoms inside the passivation materials;^[^ ^59^ ^]^ In contrast, field‐effect passivation (such as Al_2_O_3_, SiN_x_) is linked to the use of an electric field provided by fixed charges in dielectric materials.^[^ ^60^ ^]^ The porosity of the CNT film made the use of traditional passivation layers like a‐Si:H or SiN_x_ difficult. Therefore, the existing passivation techniques are not compatible with the preparation strategy of CNT/Si solar cells. Moreover, the preparation of SiO_x_ by thermal oxidation method requires a high‐temperature process, and the preparation of Al_2_O_3_ or a‐Si:H by PECVD requires high‐vacuum equipment, which restricts the further reduction of crystalline silicon cell's cost.^[^ ^61^, ^62^ ^]^ To summarize, the window‐like geometry architecture and the lack of suitable passivation techniques of CNT/Si HJ solar cells make it challenging to produce industrial‐sized cells with PCEs above 20%. Therefore, a passivation method that can perfectly match this special CNT/Si HJ is highly desirable. Such a technique should; i) have a high passivation effect at CNT/Si interface; ii) have deposition conditions compatible with the CNT/Si interface; and iii) Ideally, integratable into CNTs themselves in a composite material.

## 3 Solving CNT/Si Interface Recombination using Electrochemical Passivation

### 3.1 What is Electrochemical Passivation?

**Figure 3:**
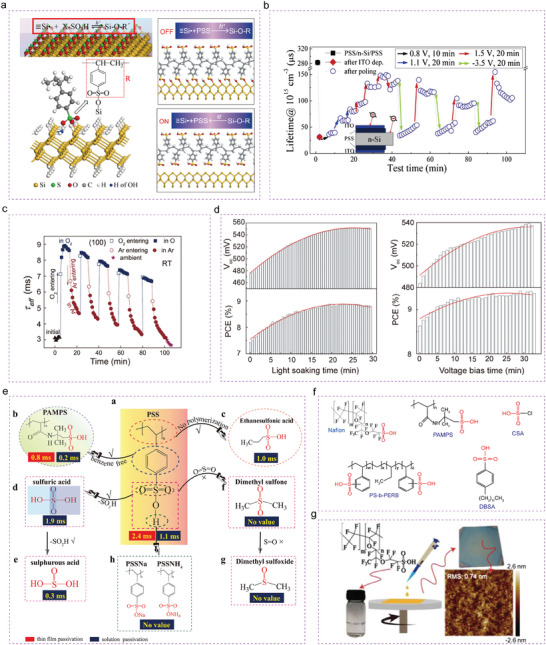
The design principle and applications of electrochemical passivation. a) The electrochemical passivation mechanism of pss.^[^ ^91^ ^]^ Copyright 2019, Elsevier for Solar Energy Material and Solar Cells. b) Electrochemically switchable behaviors of lifetime. The inset is a test structure.^[^ ^93^ ^]^ Copyright 2017, Elsevier for Electrochimica Acta. c) Evolution of lifetime of the Nafion film passivated Si wafer, measured in alternate O_2_ and Ar environments (RH:10%–20%).^[^ ^94^ ^]^ Copyright 2018, American Chemical Society for Applied Materials. d) The PV parameters of the organic‐inorganic hybrid solar cell as a function of the LS time (Left) and the forward‐bias application time (Right).^[^ ^95^ ^]^ Copyright 2018, American Chemical Society for Applied Materials. e) A functional group passivation system using a series of functional materials with a sulfonic functional group ‐SO_3_H.^[^ ^61^ ^]^ Copyright 2019, Elsevier for Solar Energy Material and Solar Cells. f) A new material system for crystalline silicon surface passivation. g) Schematic of the Nafion film‐fabrication procedure and AFM image of the Nafion film on the polished Si wafer.^[^ ^94^ ^]^ Copyright 2018, American Chemical Society for Applied Materials.

In 2017, Chen et al. proposed an organic polymer solution passivation scheme under mild conditions that was less hazardous, high‐vacuum free, achievable at low temperature, and which had a superior passivation effect to standard approaches. For example, using the polymer polystyrene sulfonate (PSS) this organic polymer passivation scheme achieved lifetimes of 28.6 ms on lightly doped (highly resistive) silicon wafers and surface recombination rates as low as 0.65 cm ^−1^s.^[^ ^91^ ^]^ The organic thin films' mechanism of passivation differs from conventional field‐effect passivation and chemical passivation, the redox reaction is shown in Equation (1):

(1) $$\equiv \mathrm{Si}\cdot +\mathrm{PSS}\underset{e^{-}}{\overset{h^{+}}{⇌}}\mathrm{Si}-O-R^{\prime }$$

where holes and electrons are denoted by *h^+^* and *e^–^*, respectively. The passivation mechanism originates from the oxidation/deoxidization process at the polymer/Si interface.^[^ ^42^ ^]^ Therefore, it is referred to as being an electrochemical passivation mechanism. Furthermore, the approach displays a ‘switching effect’ at the polymer/Si interface according to first‐principles total‐energy calculations. The polarization switching origins from the electrochemically switchable interface dipoles (SIDs) which composes by local polar regions at the polymer/silicon interface.^[^ ^92^ ^]^ The polymer molecule grafted on the H‐terminated Si surface when the Si surface acts as an electron donor, then the bond shedding when the Si surface acts as an electron acceptor, as shown in **Figure** **3**a.^[^ ^93^ ^]^

Verification of the ‘switching effect’ at the polymer/Si interface was obtained by applying external field to inject charge carriers. The geometry of the sample is shown in the inset of Figure 3b,^[^ ^93^ ^]^ where it can be seen that the lifetime increases as the positive voltage applied and lifetime drops after applied a larger negative voltage. This result further confirms the origin of electrochemical passivation for the PSS‐passivated Si surface. Furthermore, a switchable behavior of lifetime is obtained during the repetitive measurement in alternate O_2_ and N_2_ atmospheres (Figure 3c).^[^ ^94^ ^]^ This result also implies the oxidation/deoxidization passivation mechanism of the PSS thin film on Si. Meanwhile, an observant the ion of light‐induced enhancement (LIE) effect in photovoltaic performance in poly(3,4‐ethylthiophene):polystyrene sulfonate/n‐Si (PEDOT:PSS/n‐Si) hybrid solar cells is appeared (Figure 3d).^[^ ^95^ ^]^ The PEDOT:PSS/n‐Si heterojunction solar cell is a p^+^/n Schottky junction device with an internal electric field (*E*_in_) along the direction from the n‐Si to the PEDOT:PSS layer. Upon light soaking, photoexcited electron‐hole pairs are created in the Si absorber and then separated by the internal electric field. *E*_in_ (from Si to PEDOT:PSS) ensures that the holes transfer from the Si bulk to the PEDOT:PSS/Si interface, and this favours the forward reaction in Equation (1) and results in the oxidation of the Si surface. This phenomenon reduces the interface defect states and suppresses minority carrier recombination and thus increases *V*_OC_ and PCE of the hybrid solar cells. Further research revealed that the origin of the organic passivation is the sulfonic acid group on the PSS molecule.^[^ ^61^ ^]^ The passivation lifetimes by several materials are given in Figure 3e.^[^ ^61^ ^]^ It was found that a benzene ring, polymerization, or sulfonic salts do not provide effective passivation of Si. The passivation mainly originates from the functional group of ‐SO_3_H. Subsequently, Chen et al. successively discovered more than ten polymer films with high‐quality passivation effects, e.g., sulfonated polytetrafluoroethylene (Nafion), poly(2‐acrylamido‐2‐methylpropanesulfonic acid) (PAMPS), polystyrene‐block‐poly(ethylene‐ran‐butylene)‐block‐polystyrene, sulfonated, cross‐linkable (PS‐b‐PERB), etc. which established a new material system for crystalline silicon surface passivation (Figure 3f).^[^ ^61^ ^]^ Importantly, passivating thin films prepared by a simple spin‐coating method had an effective minority carrier lifetime of ≈10 ms on n‐type c‐Si wafer with a resistivity of 1–5 Ω cm, corresponding to an implied open circuit voltage (i*V*_OC_) of 724 mV in Figure 3g,^[^ ^94^ ^]^ which is a level that is in line with the a‐Si:H(i) film‐passivation scheme used in the current PV industry.

Historically, electrochemical passivation had been shown to increase the PCE of interdigitated back contact (IBC) solar cells and a PSS thin film was employed in IBC solar cells as a front surface passivation layer.^[^ ^2^ ^]^ Due to the remarkable passivation effect and antireflective coating (refraction index ≈1.5) of PSS, significant enhancement in *V*_OC_ and *J*_SC_ achieves in IBC solar cells. In addition, the shingling of solar cells has been the main module technology in the current photovoltaic industry. However, shingle solar cells are usually separated from host wafers, which leads to a decrease in solar cell efficiency due to edge recombination. This issue can also be solved by spraying coating organic passivation agents to selectively passivate silicon edge surfaces. Performing the advanced passivated edge technology on a 3 × 3 cm^2^ SHJ cell with four un‐passivated edge surfaces, an initial efficiency of 22.7% increases to a higher 24.4%.^[^ ^96^ ^]^ A shingle mini‐module by forming the cutting SHJ sub‐cells was prepared by Chen et al. and by passivating the newly formed edges with organic solution, the power of the mini‐module improved from 514.7 to 533.5 W.

### 3.2 Why is Electrochemical Passivation Suitable for Solving CNT/Si Interface Recombination?

For the high‐performance C/Si HJ solar cells, the CNT/Si interface passivation is highly desirable. As mentioned above, a passivation method should have a high passivation effect and can be deposited into CNT/Si interface as well as can integrate into CNT to be one material. Conveniently, the electrochemical passivation materials inherently allow for the fabrication of passivation layers at low temperature and high vacuum free conditions that match well with the requirements of the CNT/Si interface passivation. Moreover, some electrochemical passivation materials are good dispersants for nanotubes which can integrate passivation into CNT to be a single material. Wang et al. have demonstrated that Nafion can solubilize CNT very well, and they have successfully applied CNT/Nafion electrodes to an amperometric biosensor.^[^ ^97^ ^]^ Therefore, the *electrochemical passivation* technique will be well suited for C/Si HJ solar cells and can further boost the PCE of C/Si PV devices.

## 4 Conductive Passivating Contact Assisted CNT/Si Solar Cells Industry Evolution

**Figure 4:**
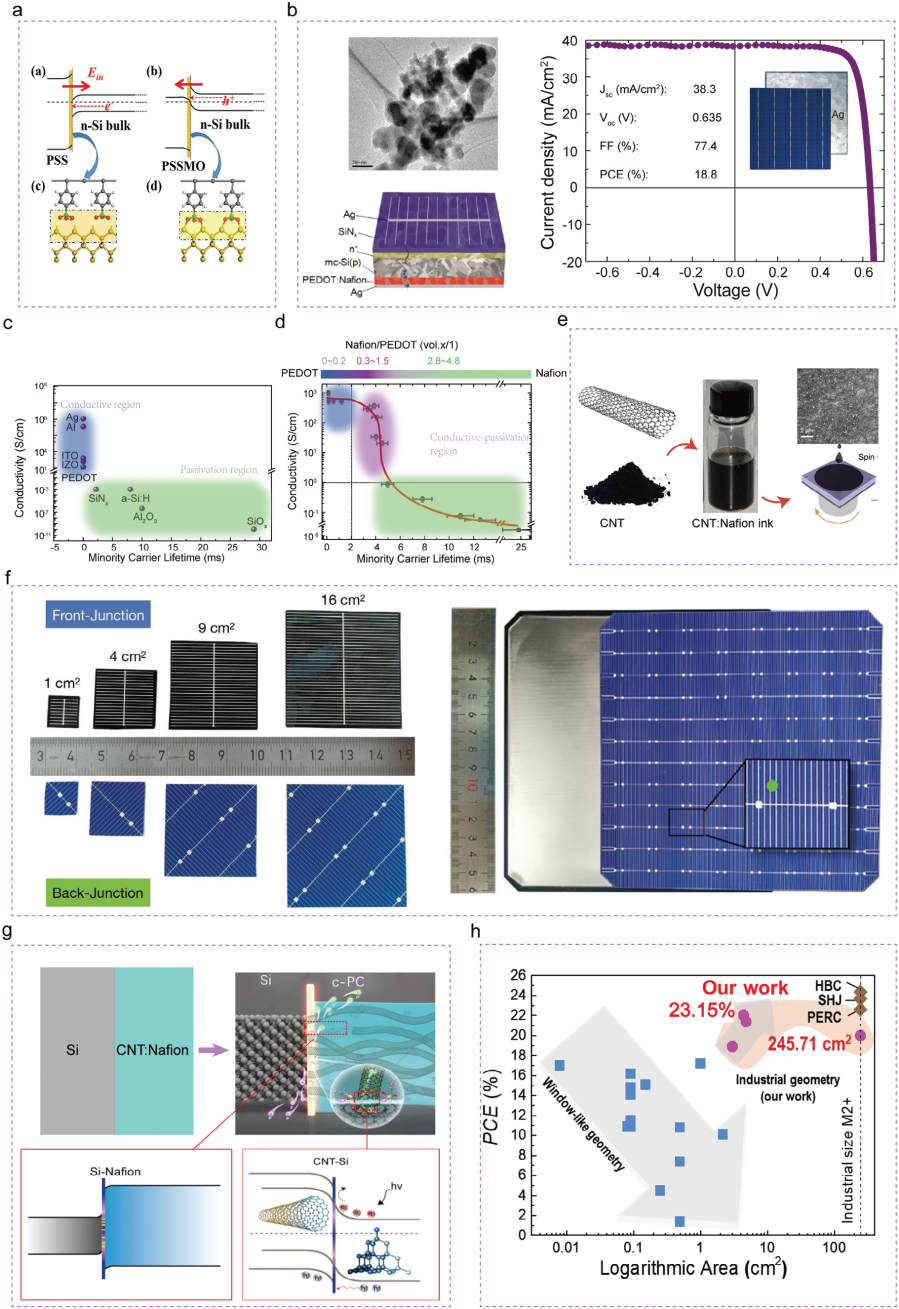
The evolution of industrial‐sized CNT/Si solar cells assisted by conductive passivation contact. a) A schematic of the band line‐up, and grafting processes for the PSS/Si interface and pssmo/si.^[^ ^100^ ^]^ Copyright 2017, John Wiley and Sons for PHYSICA STATUS SOLIDI (RRL) RAPID RESEARCH LETTERS. b) Conductive hole‐selective passivating contacts for nanostructure black Si solar cells.^[^ ^51^ ^]^ Copyright 2020, John Wiley and Sons for Advanced Functional Materials. The Functionalized phase‐like diagram of c) passivation and conductive materials, and d) passivation conductive materials.^[^ ^51^ ^]^ Copyright 2020, John Wiley and Sons for Advanced Functional Materials. e) Carbon nanotubes and Nafion are shear force mixed to form an ink that can be spin coated onto the Si wafer with an industrial size. f) Photograph of the back and front of the CNT:Nafion/Si solar cell. The back is shown before and after CNT:Nafion coating and prior to deposition of the back electrode (Ag).^[^ ^106^ ^]^ Copyright 2020, John Wiley and Sons for Advanced Functional Materials. g) The general operation principle of carbon‐based c‐PC/Si HJ solar cells. h) The selected representative results of CNT/Si HJ solar cell efficiency from the year of invention to 2022.

Electrochemical passivation presents a novel low‐cost material strategy for c‐Si surface engineering, however, some other current high‐efficiency solar cell structures, such as PERC, TOPCon, and SHJ, are not compatible with this organic passivation technology, because the electrochemical passivation materials cannot undergo high‐temperature sintering required for SiN_x_, SiO_2_, Al_2_O_3_, and other dielectric films.^[^ ^61^, ^98^ ^]^ Therefore, a new device design is required that is compatible with electrochemical passivation technology. Initially, MoO_3_ nanoparticles with high work functions were introduced into PSS to form a composite thin film, which had two functions: surface passivation and hole transport.^[^ ^99^ ^]^ As shown in **Figure** **4**a, the direction of the internal electric field (*E*_in_) was reversed for the composite PSS:MoO_x_/Si interface, which changed the deoxidation at the polymer/Si interface to oxidation at composite thin films/Si interface, corresponding to higher passivation stability.^[^ ^100^ ^]^ Then, a conductive‐passivating carrier‐selective contact was achieved using PEDOT:Nafion composite thin films. A PCE of 18.8% was reported for an industrial multi‐crystalline silicon solar cell with a back PEDOT:Nafion contact, demonstrating a solution‐processed organic passivating contact concept (Figure 4b).^[^ ^51^ ^]^ This was the first report that one material could be used to achieve both passivation and conductivity in Si PV cells and was un‐usual because as shown in Figure 4c, passivation and conductivity are normally not positively correlated with each other.^[^ ^51^ ^]^ The dielectric film materials with passivation function without a carrier transport effect, such as SiN_x_, a‐Si:H(i), Al_2_O_3_, and SiO_2_.^[^ ^62^, ^101^, ^102^ ^]^ Metals, ITO, and organic conductive polymers like poly(3,4‐ethylenedioxythiophene) (PEDOT), do not provide a passivation effect.^[^ ^28^, ^103^, ^104^ ^]^ As a result, the use of at least two materials to achieve passivation and contact strategies was always required for silicon solar cells. That leads to fabrication processes with technical complexities and a correspondingly enhanced preparation cost. In Figure 4d,^[^ ^51^ ^]^ PEDOT:Nafion, which can be considered as one material, achieved the two functions of hole selectivity and demonstrates the feasibility of a solution‐processed organic passivating contact concept. It also contributes effective back‐surface junction or field scheme for p‐type and n‐type Si solar cells, both for research purposes and as a low‐cost surface engineering strategy for future Si‐based PV technologies.

Guided by the idea of a “Conductive Passivating contact”, a simple CNT:Nafion ink combining CNTs and Nafion was developed, which enabled a low‐cost fabrication process by spin‐coating Figure 4e.^[^ ^105^ ^]^ Subsequently a front and back‐junction design of solar cells with active areas of 1–16 cm^2^ were fabricated (Figure 4f).^[^ ^106^ ^]^ In those industry‐standard solar cell geometries device, CNT:Nafion films act as a hole extraction layer and interfacial defects passivation layer simultaneously. Record maximum PCE of 15.2% and 18.9% were reported for front and back‐junction devices for 1 and 3 cm^2^ active areas, respectively. Furthermore, a PCE of 20.1% with an industrial size (245.71 cm^2^) was also achieved and this represented a breakthrough for CNT/Si solar cells in terms of area and efficiency.^[^ ^56^, ^107^, ^108^, ^109^ ^]^ In addition, a commercially available CNT soot was mixed with Nafion and used to replace the traditional back structure of PERC solar cells.^[^ ^110^, ^111^, ^112^, ^113^ ^]^ A record efficiency of 23.03% was achieved for the CNT/p‐Si solar cell.

The working principle of the CNT/Si HJ solar cell can be explained as shown in Figure 4g. The CNT with its 1D structure provides a hole‐selective contact layer and supplies the built‐in potential at the interface and Nafion is responsible for interfacial passivation. By combining carbon materials and passivation materials into an ink in this way the PV device fabrication process is dramatically simplified. These potential developments are expected to promote the Si‐based cells technology into the low‐cost strategy. Figure 4h and **Table** **2** present the results of industrial sized C/Si HJ solar cells efficiency from the year of invention to 2022. These indicate that C/Si HJ device with the C:polymer composite film demonstrated the necessary conditions of industrialization development: low cost, simple process and high efficiency.

**Table 2:** The results of industrial sized C/Si HJ solar cell efficiency from the year of invention to 2022.

Solar cell structure	PCE [%]	Active area [cm^2]^	*V*_oc_ [mV]	*J*_sc_ [mA cm^−2^]	FF [%]	Year	First Institution
Graphene/Si	18.8	5.5	654	40.1	71.9	2022	Hebei University^[^ ^114^ ^]^
CNT/Si	18.9	3	631	38.8	77.2	2019	Karlsruhe Institute of Technology^[^ ^106^ ^]^
	21.4	4.8	654	39.9	82	2020	Hebei University and Karlsruhe Institute of Technology ^[^ ^105^ ^]^
	20.1	245.71	646	39.5	78.9	2020	Hebei University and Karlsruhe Institute of Technology ^[^ ^105^ ^]^
	22.04	4.4	683.4	40.38	79.9	2021	Hebei University and Karlsruhe Institute of Technology ^[^ ^115^ ^]^
	22.04		669.4	40.3	81.7	2022	Hebei University and Karlsruhe Institute of Technology ^[^ ^116^ ^]^
	23.03	6.2	679.4	41.3	82.1	2022	Hebei University and Karlsruhe Institute of Technology ^[^ ^117^ ^]^

## 5 Perspectives

### 5.1 The Key Challenges and Developing Trends of C/Si HJ Solar Cells

The state‐of‐the‐art C/Si HJ solar cell has been a promising field and the PCE has reached over 23% with an astonishing speed. However, several challenges remain for C/Si HJ solar cells. For instance, whether or not it is a p‐n junction or a Schottky junction at the heart of a C/Si HJ has not been completely clarified, and this is mainly due to the complexity and diversity of the carbon films. Particularly, for the unsorted‐SWCNT/Si HJ, it was demonstrated that the m‐SWCNTs played a very significant role. In the future, pure semiconducting or metallic C films with high purity and quality are required to understand the nature of C/Si HJs and their functions in solar cells.

**Figure 5:**
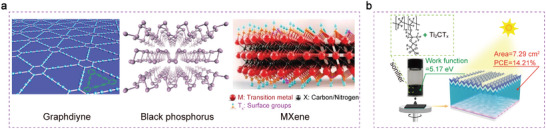
a) Some materials of low‐dimensional family.^[^ ^123^ ^]^ Copyright 2014, Springer Nature for Nature Nanotechnology. b) MXene:Nafion–Si HJ solar cell with a PCE of 14.21% at the area of 7.29 cm^2^.^[^ ^124^ ^]^ Copyright 2022, John Wiley and Sons for Solar RRL.

In addition, the most commonly used carbon materials in C/Si HJ are a‐C, graphite, graphene, fullerene, and CNTs.^[^ ^118^, ^119^, ^120^, ^121^ ^]^ In fact, other forms of carbon or carbon‐like materials such as graphdiyne,^[^ ^119^ ^]^ carbon black,^[^ ^120^ ^]^ carbon soot,^[^ ^118^ ^]^ black phosphorus,^[^ ^121^ ^]^ or 2D transition metal carbon/nitrides (MXenes),^[^ ^122^ ^]^ also have the capacity to form high performance Si solar cells (**Figure** **5**a). Chen et al. have demonstrated an effect of termination by Nafion to Ti_2_CT_x_ MXene groups (MXene:Nafion). Nafion doping dramatically enhanced the work function of the Ti_2_CT_x_ MXene from 3.96 to 5.17 eV. Moreover, MXene:Nafion layer achieved the functions of defect passivation and carrier selective transmission simultaneously, leading to a MXene:Nafion–Si HJ solar cell with a PCE of 14.21% at the area of 7.29 cm^2^ (Figure 5b).

### 5.2 Device Structure

**Figure 6:**
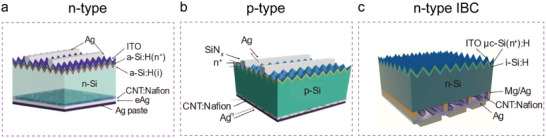
CNT/Si with different solar cell structures. a) CNT/n‐Si HJ solar cell.^[^ ^116^ ^]^ Copyright 2021, John Wiley and Sons for Advanced Science. b) CNT/p‐Si HJ solar cell.^[^ ^117^ ^]^ Copyright 2022, Elsevier for Carbon. c) IBC‐CNT solar cell design.^[^ ^127^ ^]^ Copyright 2023, John Wiley and Sons for Small Structures.

As shown in Figure 4f, industry standard device geometries were fabricated with the use of CNT:Nafion ink. This led to the formation of a hybrid nanotube/Nafion passivated charge selective contact, and solar cells with high PCE and industry active areas were fabricated but the structure of the CNT/Si HJ solar cell still needs to be optimized. As shown in **Figure** **6**a,b, the single‐step deposition of a CNT:Nafion layer is analogous to a‐Si:H(p)/a‐Si:H(i) or SiNx/Al_2_O_3_/c‐Si(p+) layer stacks. That means that CNT:Nafion not only acts as the p type emitter of n‐Si devices but that it can also serve as a back surface field of p‐Si HJ solar cells. However, the front side architecture of the C/Si HJ solar cells have to date always been based on SHJ or PERC.^[^ ^94^, ^105^ ^]^ The ultimate advancement of the DASH concept will be to develop n‐type CNT inks to complement the existing p‐type inks and these will allow for truly low temperature and cheap all carbon contacted CNT(p)/n‐Si/CNT(n^+^) cells to be built.

In addition, the IBC structure is the current state‐of‐art device geometry in photovoltaics^[^ ^125^, ^126^ ^]^ where the emitter and contact are on the back of the Si substrate. This provides numerous advantages over conventional geometries: zero optical shading, independent control for optimum optical performance in the illuminating front side and optimum electrical performance with simpler interconnection techniques on the back side, consequently higher efficiencies, and improved aesthetics. Figure 6c is an IBC‐CNT device concept, which has been demonstrated in our recent work. Ideally, a photolithographic‐free, room‐temperature processes, large‐area (4.76 cm^2^) and highly efficient (17.53%) IBC‐CNT solar cell design was obtained. A suns‐*V_OC_* measurement was employed to evaluate the pseudo *FF* (*pFF*). It was found that a *pFF* of up to 81.33% and an efficiency exceeding 21.39% was obtained for this kind of simple IBC‐CNT cells. Further improvement for the IBC‐CNT performance may be possible by developing efficient low work function electron‐selective contacts that are compatible with the current processes.

### 5.3 Manufacturing Costs Potential

In all of the C/Si HJ solar cells mentioned above, the PCE and active area of the CNT/Si HJ solar cells has been greatly improved by using a “low‐dimensional nanomaterials + organic passivation” strategy whilst at the same time reducing the complexity of fabrication in a CNT/Si HJ solar cell. The standard in‐line process for the rear structure of industrial PERC solar cells (22.52%) contains four steps. These requires high temperature and vacuum equipment such as ALD (100‐350 °C, 10^−5^ Pa), PECVD (300‐450 °C), laser instrument and metallization (firing at 800 °C), while the process for the rear structure of p‐Si solar cells (23.03%) in CNT/Si solar cells only require two steps, which do not require energy‐intensity equipment and can be prepared by spin‐coating at room temperature and at atmospheric pressure.

More importantly, the state‐of‐the‐art CNT/Si HJ solar cells use commercially available raw SWCNT soot and Nafion to develop a single conductive passivating contact.^[^ ^110^, ^111^, ^128^ ^]^ Two types of SWCNT raw soot with difference costs have been used under the names AP‐SWCNT and SG65i.^[^ ^110^ ^]^ AP‐SWCNTs have an average diameter of 1.4 nm and a carbonaceous purity of 60–70% which is cheaper than that of SG‐65i with an average diameter of 0.78 nm and purity of 95%. The AP‐SWNTs had a PCE of 22.04%, and the SG65i solar cells performed better in all parameters with a PCE of 23.03%. The results show that SWCNTs without high purity can also achieve high efficiency, yet the device shows higher performance as the purity increases. A trade‐off between the PCE and cost will be consistent with high performance Si solar cells used in the current PV industry.

The main cost of the current C/Si HJ devices maybe the metal electrode cost and the passivation solution cost (such as Nafion). To reduce the use of silver paste, other low‐priced metal such as Cu was evaporated as the electrode of the C/Si HJ solar cells. Moreover, some previous works reported that the work functions of Ag and Cu are 4.26 eV and 4.65 eV, respectively, which indicates that Cu is more suitable as the electrode.^[^ ^129^ ^]^ Using this approach we have achieved decent PCE and further optimization is under way. On the other hand, for the industrialization of the C/Si HJ solar cells, developing cheaper passivation solutions to form cheaper passivating conductive inks are needed to be further explored.

### 5.4 Absorption Spectrum

**Figure 7:**
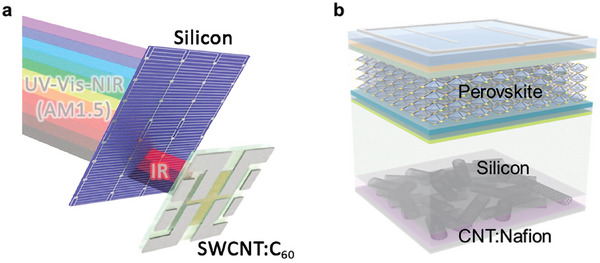
a) Schematic diagram of the 4‐terminal tandem solar cell formed by a bifacial Si solar cell and an SWCNT/C_60_ organic cell.^[^ ^55^ ^]^ Copyright 2021, Elsevier for Carbon. b) Schematic diagram of a perovskite‐C/Si two terminal tandem device.

According to the theoretical “roll‐up” of a graphene lattice, SWCNT have first (S_11_) and second (S_22_) optical transitions ranging from 2.57 eV (visible) to 0.5 eV (near‐infrared) and these have been proposed as a promising candidates to enhance absorption spectrum of existing photovoltaic materials.^[^ ^130^ ^]^ Wieland et al. coupled a bifacial Si solar cell to an SWCNT/C_60_ organic cell in a 4‐terminal stack to extend the IR‐absorption of Si devices (**Figure** **7**a).^[^ ^55^ ^]^ It was found that (7,6) SWCNTs are currently the best choice, and an additional 0.156 mA cm^−2^ (value obtained from integration over 300–1500 nm) can be expected. However, the mismatch in current density from the SWCNT/C_60_ and Si tandem solar cell need to be further optimization. In the future, increasing the internal reflectance at back interface, reducing the absorption of the window layer in the long wavelength range, SWCNT based/Si tandem solar cells are more likely to lead to performance gains in this spectral region.

### 5.5 Perovskite/Carbon‐Si HJ Tandem Solar Cells

Perovskite/silicon tandem solar cells (PK/Si TSCs) are currently pushing the PCE beyond the single‐junction Shockley‐Queisser (SQ) limit.[^131^, ^132^, ^133^, ^134^, ^135^, ^136^, ^137^, ^138^, ^139^, ^140^, ^141^ ^]^ Recently, PK/Si TSC obtained a PCE of 33.9%,^[^ ^142^ ^]^ however, the preparation process of the tandem device was quite complex.^[^ ^134^, ^143^, ^144^, ^145^, ^146^, ^147^, ^148^, ^149^ ^]^ At present, most of the high‐efficiency PK/Si TSCs select.^[^ ^141^, ^150^, ^151^, ^152^, ^153^, ^154^ ^]^ The preparation process of SHJ sub‐cell is associated with technological complexities and expensive vacuum equipment, which does not match the solution preparation route of perovskite top sub‐cell. C/Si HJ solar cells can be fabricated at room‐temperature, vacuum‐free environment and potentially cost‐effective by simplifying processes and equipment. The manufacture technology of C/Si solar cell is fully compatible with the top perovskite preparation process. In this way, the perovskite‐C/Si tandem solar cells, as shown in Figure 7b, contributes a great potential both for research purposes and as a low‐cost strategy for future Si‐based PV technologies. Moreover, Chen et al. also found a significant improvement of the infrared light management (1000‐1200 nm) in the CNT/Si solar cell.^[^ ^155^ ^]^ The improvement may benefit of rear surface chemical polishing and low refractive index of the back CNT/Si layer. For perovskite‐Si tandem solar cells, Si solar cell is mainly absorbing long wavelength light (740‐1200 nm). So, the increased of the infrared light management of CNT/Si solar cell maybe open a new way for further enhancing the current of perovskite‐Si tandem solar cells.

### 5.6 Stability

The long‐term stability of “CNT‐organic passivation” composite layer is often a concern for researchers wishing to use this process. CNTs have excellent stability in ambient, humid, hot, or ultraviolet radiation conditions,^[^ ^156^, ^157^, ^158^, ^159^ ^]^ but the hygroscopic nature of the organic passivation materials (such as PSS, Nafion) are known to lead to performance reductions^[^ ^160^ ^]^ and these must be addressed. In fact, humidity is the main limitation for the stability of organic passivation film. Chen et al. has demonstrated that the electrochemical passivation effect was increased with both oxygen and light conditions, while lifetime decreased under high‐humidity atmosphere. For the thermal stability, Yang et al. present that organic passivation thin film maintains a superb passivation effect with the annealed temperatures up to 200 °C and hardly presents a passivation effect with the annealing beyond 250 °C. The thermal behavior of organic passivation thin film is similar to the industry's champion passivation material hydrogenated amorphous silicon (a‐Si:H).^[^ ^161^ ^]^

**Figure 8:**
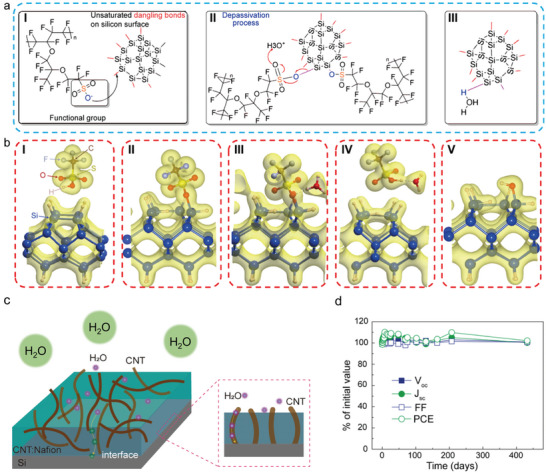
The long‐term stability of C/Si solar cells. a) Schematic diagram of the chemical reaction of the passivation and de‐passivation mechanism.^[^ ^162^ ^]^ Copyright 2022, John Wiley and Sons for Advanced  Energy and Sustainability Research. b) DFT calculated charge density isosurfaces (0.05 e Bohr‐3) for the passivation and de‐passivation process.^[^ ^162^ ^]^ Copyright 2022, John Wiley and Sons for Advanced Energy and Sustainability Research. c) Physiochemical mechanism of the CNT/Nafion and Si interfaces. d) Stability of CNT/Si HJ solar cell with encapsulation, PV performance values were found to be stable over a period of 400 day in ambient conditions.^[^ ^115^ ^]^ Copyright 2021, John Wiley and Sons for Advanced Science.

To clarify the role of water molecules, density functional theory (DFT) was used to reveal the passivation process. The chemical reaction of the passivation or de‐passivation process is shown in **Figure** **8**a I–III. The passivation process is illustrated in Figure 8a‐I. Nafion, an organic polymer with a passivation functional group, neutralizes the unsaturated dangling bonds on the Si surface. As the H_3_O^+^ approaches the passivation interface, it triggers a de‐passivation process as shown in Figure 8a‐II. Figure 8a‐III demonstrates that the passivation bond is broken, allowing Si to combine with OH in the solution, resulting in failed passivation. Figure 8b I–V presents the DFT calculated charge density isosurfaces (0.05 e Bohr‐3) for the passivation and de‐passivation process. According to the reaction calculation, H and O atoms of Nafion bond with the surface Si atom results in the total energy decrease of the Nafion‐Si surface (Figure 8b‐I,II), which indicates a passivation process. However, the passivation fails when H_3_O^+^ is approaching the interface of Nafion‐Si (Figure 8b–III). Moreover, the surface Si atom will bond with OH^−^ in solution to balance the hanging bond of the Si surface (Figure 8b–V). These results demonstrate the role of water molecules in the passivation and de‐passivation process. Figure 8c shows the CNT: organic composite film with the water molecules, the CNTs provides an additional path for water molecules to the CNT/Si interface, promoting the instability. Therefore, the instability of the organic passivated CNT/Si complex interface is despite both the organic passivation layer and CNTs. However, as final solar cells are always encapsulated in a PV module, so with a simple encapsulation, a more 400 days stable photovoltaic performance is demonstrated (Figure 8d).

### 5.7 Scientific Directions

**Figure 9:**
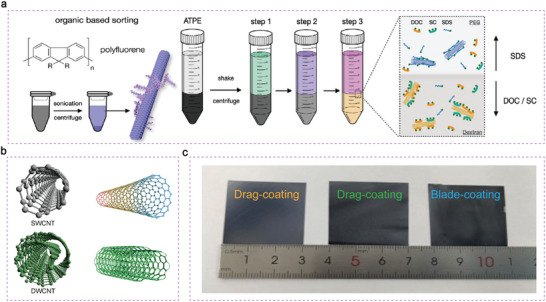
Scientific directions of C/Si HJ solar cells. a) Extraction of chiral species using polymer wrapping in organic solvents and in aqueous with surfactants in a two‐phase extraction process (ATPE).^[^ ^60^ ^]^ Copyright 2020, John Wiley and Sons for Advanced Energy Materials. b) The schematic diagram of SWCNT and DWCNT. c) The different methods for preparation of C/Si solar cells with larger active areas.

CNT/Si solar cells have seen a dramatic and encouraging improvement in recent years,^[^ ^107^, ^163^, ^164^, ^165^, ^166^, ^167^ ^]^ and this is despite the use of a polychiral mixture of CNTs as found in the raw materials. A further step in this field will rely upon a clarification of the working mechanism and interface structure of the HJ. Precise energy band engineering and will requires CNT materials with a narrow structural distribution and fortunately. Preparing high‐purity CNTs with well‐controlled structures and properties has been one of the most active topic in the community of CNT (**Figure** **9**a).^[^ ^56^ ^]^ Intense efforts have been made to achieve selective synthesis of CNTs with uniform electronic type or specific (n, m) species. Selective synthesis depends on controlling the synthesis process thermodynamically and catalyst design.^[^ ^168^ ^]^ However, either the purity or yield of selective synthesized CNTs cannot fulfill the requirement of applications for the moment. On the other hand, post‐synthesis methods to separate CNTs by their structures have been developed and are more attractive.^[^ ^161^ ^]^ Liquid phase separation methods have achieved separation of s‐CNTs and m‐CNTs as well as single‐chirality separation of dozens of (n, m) species with diameter from 0.75 to 1.41 nm.^[^ ^169^ ^]^ High resolution and scalability of liquid phase separation grant the separated CNTs a great promise in applications specifically in the field of photovoltaics.^[^ ^170^ ^]^ However, molecular recognition of the abovementioned methods stems from selective coating of surfactants or polymer on highly dispersed CNTs, implying the necessity of surface coating for both dispersing and separation of CNTs. These molecules coating on the sidewalls of nanotubes may significantly affect the properties of CNT.^[^ ^171^ ^]^ Hence, completely removal of surface coating is the prerequisite for applications.

Additionally, DWCNTs were also separated by electronic type of outer and inner nanotubes through liquid phase separation.^[^ ^172^, ^173^ ^]^ Though composed of two SWCNTs, DWCNTs is not a simple combination of two SWCNTs, considering the interaction between the two sidewalls (Figure 9b). Coupling of the sidewalls leads to some new phenomena such as superconductivity,^[^ ^174^ ^]^ shifted optical modes,^[^ ^175^ ^]^ higher mechanical and chemical stability.^[^ ^175^ ^]^ DWCNTs posse a wider optical absorption range than SWCNTs due to different diameter distribution of inner and outer nanotubes. Besides, DWCNTs are reported to be easily dispersed comparing with SWCNTs and are perfect candidate for solution‐processable transparent CNT electrodes.^[^ ^176^, ^177^ ^]^ These properties suggest that DWCNTs have a great potential in photovoltaics.^[^ ^77^ ^]^ Yet, DWCNT‐based photovoltaic devices, especially DWCNT/Si solar cells are seldom reported at the moment.

Finally, the efficiency of the C/Si devices is relatively higher, to realize the commercialization of C/Si solar cells, scale‐up the manufacturing of C/Si HJ solar cells also needs to be consideration. The most reported high efficiency C/Si solar cells were prepared by spinning‐coating method. However, it is well known that spin‐coating method is probably limited for large‐area (>10 cm^2^) coating.^[^ ^178^ ^]^ Other coating processes in Figure 9c, such as drag‐coting,^[^ ^179^ ^]^ blade coating,^[^ ^180^ ^]^ wire‐bar (D‐bar) coating, slot‐die coating,^[^ ^181^ ^]^ meniscus coating,^[^ ^182^ ^]^ spray coating,^[^ ^183^ ^]^ inkjet printing,^[^ ^184^, ^185^, ^186^, ^187^ ^]^ and screen printing^[^ ^188^ ^]^ can expand the area limit compared with the spin‐coating method. Those methods proves that CNT:Nafion films can be applied to industrialization. Furthermore, careful investigation of the solar cell module and encapsulation will also be the main aims in the future.

## 6 Conclusion

In summary, C/Si HJ solar cells provide an avenue towards low‐cost but high efficiency photovoltaics. However, due to the inhomogeneities of the carbon‐based film during fabrication and the lack of interface passivation, the PCE and active areas are lower than C/Si solar cells. Electrochemical passivation technology solves the C/Si interface passivation using a low‐cost solution method. Moreover, by mixing low‐dimensional semiconductors in the precursor organic passivation solution for an ink, one material that possesses both a good passivation and conductivity, which simplifies the cell process. CNT/Si HJ solar cells achieves the highest PCE over 23%, which is comparable to industrial efficiency. Furthermore, by employing the passivation contact ink, the preparative technique of C/Si solar cells is from the window‐like geometry fabrication to the large‐scale manufacture (the device area with industrial size of 245.71 cm^2^). However, as mentioned above, the future development of solar cells should focus on the efficiency, costs, tandem devices application, stability and so on. These potential developments are expected to promote the C/Si cells approach into the realm of competitive c‐Si cell technology.

## Conflict Of Interest

The authors declare no conflict of interest.

## References

[1] M. Green , E. Dunlop , J. Hohl‐Ebinger , M. Yoshita , N. Kopidakis , X. Hao , Prog. Photovoltaics 2021, 29, 15.

[2] J. Chen , Y. Shen , B. Chen , K. Ge , J. Guo , Z. Wang , F. Li , Y. Xu , Y. Mai , Sol. RRL 2017, 1, 1700079.

[3] M. Raghuwanshi , M. Chugh , G. Sozzi , A. Kanevce , T. D. Kühne , H. Mirhosseini , R. Wuerz , O. Cojocaru‐Mirédin , Adv. Mater. 2022, 34, 2203954. 10.1002/adma.202203954 35900293

[4] J. Britt , C. Ferekides , Appl. Phys. Lett. 1993, 62, 2852.

[5] K. Fukuda , K. Yu , T. Someya , Adv. Energy Mater. 2020, 10, 2000765.

[6] Y. Zhao , F. Ma , Z. Qu , S. Yu , T. Shen , H.‐X. Deng , X. Chu , X. Peng , Y. Yuan , X. Zhang , J. You , Science 2022, 377, 534. 10.1126/science.abp8873 35901131

[7] M. A. Green , Y. Hishikawa , W. Warta , E. D. Dunlop , D. H. Levi , J. Hohl‐Ebinger , A. W. H. Ho‐Baillie , Prog. Photovoltaics 2017, 25, 676.

[8] M. A. Green , Prog. Photovoltaics 2009, 17, 183.

[9] J. Mandelkorn , J. H. Lamneck , J. Appl. Phys. 1973, 44, 4787.

[10] J. Mandelkorn , J. H. Lamneck , Sol. Cells 1990, 29, 130.

[11] M. A. Green , Joule 2019, 3, 633.

[12] K. H. Kim , C. S. Park , J. D. Lee , J. Y. Lim , J. M. Yeon , I. H. Kim , E. J. Lee , Y. H. Cho , Jpn. J. Appl. Phys. 2017, 56.

[13] J. Zhao , A. Wang , M. A. Green , Prog. Photovoltaics 1999, 7, 474.

[14] D. D. Smith , P. Cousins , S. Westerberg , R. De Jesus‐Tabajonda , G. Aniero , Yu‐C Shen , IEEE J. Photovoltaics 2014, 4, 1469.

[15] D. Adachi , J. L. Hernández , K. Yamamoto , Appl. Phys. Lett. 2015, 107.

[16] K. Masuko , M. Shigematsu , T. Hashiguchi , D. Fujishima , M. Kai , N. Yoshimura , T. Yamaguchi , Y. Ichihashi , T. Mishima , N. Matsubara , T. Yamanishi , T. Takahama , M. Taguchi , E. Maruyama , S. Okamoto , N. Matsubara , IEEE J. Photovoltaics 2014, 4, 1433.

[17] R. Chen , K. Sun , Qi Zhang , Y. Zhou , M. Li , Y. Sun , Z. Wu , Y. Wu , X. Li , J. Xi , C. Ma , Y. Zhang , J. Ouyang , J. Xi , IScience 2019, 12, 66. 30677740 10.1016/j.isci.2019.01.003 PMC6352564

[18] T. G. Allen , J. Bullock , X. Yang , A. Javey , S. De Wolf , Nat. Energy 2019, 4, 928.

[19] C.‐H. Hsu , C.‐W. Huang , Y.‐S. Cho , W‐Yu Wu , D.‐S. Wuu , X.‐Y. Zhang , W.‐Z. Zhu , S.‐Y. Lien , C.‐S. Ye , Surf. Coat. Technol. 2019, 358, 975.

[20] J. Schmidt , F. Werner , B. Veith , D. Zielke , S. Steingrube , P. P. Altermatt , S. Gatz , T. Dullweber , R. Brendel , Energy Procedia 2012, 15, 39.

[21] M. B. Aksari , A. Eray , Energy Procedia 2011, 10, 105.

[22] X. Liu , Y. Ji , Z. Lu , Y. Sun , H. Yang , J. Liu , Y. Zhang , D. Li , Y. Cao , W. Li , J. Xu , K. Chen , Phys. E 2020, 120.

[23] H. Lin , M. Yang , X. Ru , G. Wang , S. Yin , F. Peng , C. Hong , M. Qu , J. Lu , L. Fang , Nat. Energy 2023, 1.

[24] H. Q. C. G. Markus , Fischer 2021.

[25] Z. Xia , P. Li , Y. Liu , T. Song , Q. Bao , S.‐T. Lee , B. Sun , Nano Res. 2017, 10, 3856.

[26] A. H. M. Smets , W. M. M. Kessels , M. C. M. Van De Sanden , Appl. Phys. Lett. 2003, 82, 1547.

[27] Y. Liu , Y. Li , Y. Wu , G. Yang , L. Mazzarella , P. Procel‐Moya , A. C. Tamboli , K. Weber , M. Boccard , O. Isabella , X. Yang , B. Sun , Materials Science and Engineering: R: Reports 2020, 142, 100579.

[28] H. Shi , C. Liu , Q. Jiang , J. Xu , Adv. Electron. Mater. 2015, 1, 1500017.

[29] L. V. Kayser , D. J. Lipomi , Adv. Mater. 2019, 31, 1806133. 10.1002/adma.201806133 PMC6401235 30600559

[30] W. Wang , H. Lin , Z. Yang , Z. Wang , J. Wang , L. Zhang , M. Liao , Y. Zeng , P. Gao , B. Yan , J. Ye , B. Yan , IEEE J. Photovoltaics 2019, 9, 1120.

[31] L. G. Gerling , S. Mahato , A. Morales‐Vilches , G. Masmitja , P. Ortega , C. Voz , R. Alcubilla , J. Puigdollers , Sol. Energy Mater. Sol. Cells 2016, 145, 115.

[32] Y. Shao , G. C. Bazan , A. J. Heeger , Adv. Mater. 2007, 19, 370.

[33] Y. Zhang , F. Zu , S.‐T. Lee , L. Liao , Ni Zhao , B. Sun , Adv. Energy Mater. 2014, 4, 1300923.

[34] J. He , P. Gao , Z. Yang , J. Yu , W. Yu , Yu Zhang , J. Sheng , J. Ye , J. C. Amine , Yi Cui , Adv. Mater. 2017, 29, 1606321. 10.1002/adma.201606321 28151568

[35] D. Zielke , C. Niehaves , W. Lövenich , A. Elschner , M. Hörteis , J. Schmidt , Energy Procedia 2015, 77, 339.

[36] X. Yang , Y. Lin , J. Liu , W. Liu , Q. Bi , X. Song , J. Kang , F. Xu , L. Xu , M. N. Hedhili , D. Baran , X. Zhang , T. D. Anthopoulos , S. De Wolf , Adv. Mater. 2020, 32, 2002608. 10.1002/adma.202002608 32613655

[37] J. Dréon , Q. Jeangros , J. Cattin , J. Haschke , L. Antognini , C. Ballif , M. Boccard , Nano Energy 2020, 70.

[38] J. Bullock , M. Hettick , J. Geissbühler , A. J. Ong , T. Allen , C. M. Sutter‐Fella , T. Chen , H. Ota , E. W. Schaler , S. De Wolf , C. Ballif , A. Cuevas , A. Javey , S. De Wolf , Nat. Energy 2016, 1, 7.

[39] H. Zhu , J. Wei , K. Wang , D. Wu , Sol. Energy Mater. Sol. Cells 2009, 93, 1470.

[40] X. Li , Z. Lv , H. Zhu , Adv. Mater. 2015, 27, 6574. 10.1002/adma.201502999 26422457

[41] H. Mehmood , T. Tauqeer , IET Circuits, Devices & Systems 2017, 11, 675.

[42] G. K. Bhagavat , K. D. Nayak , Thin Solid Films 1979, 64, 62.

[43] H. A. Yu , Y. Kaneko , S. Yoshimura , S. Otani , Appl. Phys. Lett. 1996, 68, 549.

[44] W. Gong , P. Wang , D. Dai , Z. Liu , L. Zheng , Y. Zhang , J. Mater. Chem. C 2021, 9, 3025.

[45] H.‐M. Dong , Y.‐F. Duan , F. Huang , J.‐L. Liu , Front. Phys. 2018, 13, 5.

[46] X. Kong , L. Zhang , B. Liu , H. Gao , Y. Zhang , H. Yan , X. Song , RSC Adv. 2019, 9, 877. 10.1039/c8ra08035f PMC9059660 35517633

[47] J. Jing , S. Dong , K. Zhang , B. Xie , J. Zhang , Yu Song , F. Huang , Nano Energy 2022, 93, 106814.

[48] T. Maruyama , S. Naritsuka , Carbon Nanotubes‐Synthesis, Characterization, Applications 2011, 46.

[49] T. W. Odom , J.‐L. Huang , P. Kim , C. M. Lieber , Nature 1998, 391, 64.

[50] S. M. Bachilo , M. S. Strano , C. Kittrell , R. H. Hauge , R. E. Smalley , R. B Weisman , Science 2002, 298, 2366. 12459549 10.1126/science.1078727

[51] Lu Wan , C. Zhang , K. Ge , X. Yang , F. Li , W. Yan , Z. Xu , L. Yang , Y. Xu , D. Song , J. Chen , D. Song , Adv. Energy Mater. 2020, 10, 1903851.

[52] E. Kymakis , I. Alexandrou , G. A. J. Amaratunga , G. Amaratunga , J. Appl. Phys. 2003, 93, 1768.

[53] B. Xu , J. Yin , Z. Liu , S. Suzuki , Physical and Chemical Properties of Carbon Nanotubes 2013, 383.

[54] G. Pennington , N. Goldsman , Phys. Rev. B 2005, 71, 205318.

[55] L. Wieland , C. Rust , H. Li , M. Jakoby , I. Howard , F. Li , J. Shi , J. Chen , B. S. Flavel , Carbon 2021, 184, 835.

[56] L. Wieland , H. Li , C. Rust , J. Chen , B. S. Flavel , Adv. Energy Mater. 2021, 11, 2002880.

[57] D. D. Tune , B. S. Flavel , R. Krupke , J. G. Shapter , Adv. Energy Mater. 2012, 2, 1055.

[58] E. Muramoto , Y. Yamasaki , F. Wang , K. Hasegawa , K. Matsuda , S. Noda , RSC Adv. 2016, 6, 93581.

[59] H. Mehmood , H. Nasser , T. Tauqeer , R. Turan , Int. J. Energy Res. 2020, 44, 10753.

[60] P. Saint‐Cast , J. Benick , D. Kania , L. Weiss , M. Hofmann , J. Rentsch , R. Preu , S. W. Glunz , IEEE Electron Device Lett. 2010, 31, 697.

[61] J. Chen , K. Ge , B. Chen , J. Guo , L. Yang , Yu Wu , G. Coletti , H. Liu , F. Li , D. Liu , Z. Wang , Y. Xu , Y. Mai , D. Liu , Sol. Energy Mater. Sol. Cells 2019, 195, 105.

[62] S. De Wolf , M. Kondo , Appl. Phys. Lett. 2007, 90.

[63] H. A. Yu , T. Kaneko , S. Yoshimura , Y. Suhng , S. Otani , Y. Sasaki , Appl. Phys. Lett. 1996, 69, 4080.

[64] Z. Q. Ma , B. X. Liu , Sol. Energy Mater. Sol. Cells 2001, 69, 344.

[65] K. Kita , C. Wen , M. Ihara , K. Yamada , J. Appl. Phys. 1996, 79, 2800.

[66] K. L. Narayanan , M. Yamaguchi , Appl. Phys. Lett. 1999, 75, 2107.

[67] K. L. Narayanan , M. Yamaguchi , H. Azuma , Appl. Phys. Lett. 2002, 80, 1287.

[68] M. H. Yun , J. W. Kim , S. Yi Park , D. S. Kim , B. Walker , J. Y. Kim , J. Mater. Chem. A 2016, 4, 16417.

[69] X. Li , H. Zhu , K. Wang , A. Cao , J. Wei , C. Li , Yi Jia , Z. Li , X. Li , D. Wu , Adv. Mater. 2010, 22, 2748.

[70] X. Li , L. Fan , Z. Li , K. Wang , M. Zhong , J. Wei , D. Wu , H. Zhu , Adv. Energy Mat. 2012, 2.

[71] X. Miao , S. Tongay , M. K. Petterson , K. Berke , A. G. Rinzler , B. R. Appleton , A. F. Hebard , Nano Lett. 2012, 12, 2750. 10.1021/nl204414u 22554195

[72] X. Li , D. Xie , H. Park , T. H. Zeng , K. Wang , J. Wei , M. Zhong , D. Wu , J. Kong , H. Zhu , Adv. Energy Mater. 2013, 3, 1034.

[73] H. Liu , D. Nishide , T. Tanaka , H. Kataura , Nat. Commun. 2011, 2, 309. 21556063 10.1038/ncomms1313 PMC3113293

[74] E. Shi , H. Li , L. Yang , L. Zhang , Z. Li , P. Li , Y. Shang , S. Wu , X. Li , J. Wei , K. Wang , H. Zhu , D. Wu , Y. Fang , A. Cao , J. Wei , Nano Lett. 2013, 13, 1781. 10.1021/nl400353f 23517083

[75] Y. Song , X. Li , C. Mackin , Xu Zhang , W. Fang , T. Palacios , H. Zhu , J. Kong , Nano Lett. 2015, 15, 2110. 10.1021/nl505011f 25685934

[76] J. Ma , He Bai , W. Zhao , Y. Yuan , K. Zhang , Sol. Energy 2018, 160, 84.

[77] J. Wei , Yi Jia , Q. Shu , Z. Gu , K. Wang , D. Zhuang , G. Zhang , Z. Wang , J. Luo , A. Cao , D. Wu , A. Cao , Nano Lett. 2007, 7, 2321. 10.1021/nl070961c 17608444

[78] Yi Jia , J. Wei , K. Wang , A. Cao , Q. Shu , X. Gui , Y. Zhu , D. Zhuang , G. Zhang , B. Ma , L. Wang , W. Liu , Z. Wang , J. Luo , D. Wu , B. Ma , Adv. Mater. 2008, 20, 4598.

[79] Z. Li , V. P. Kunets , V. Saini , Y. Xu , E. Dervishi , G. J. Salamo , A. R. Biris , ACS Nano 2009, 3, 1414. 10.1021/nn900197h 19456166

[80] P. Wadhwa , Bo Liu , M. A. Mccarthy , Z. Wu , A. G. Rinzler , Nano Lett. 2010, 10, 5005. 10.1021/nl103128a 21047122

[81] Y. Jia , P. Li , X. Gui , J. Wei , K. Wang , H. Zhu , D. Wu , L. Zhang , A. Cao , Y. Xu , Appl. Phys. Lett. 2011, 98.

[82] E. Shi , L. Zhang , Z. Li , P. Li , Y. Shang , Yi Jia , J. Wei , K. Wang , H. Zhu , D. Wu , S. Zhang , A. Cao , D. Wu , Sci. Rep. 2012, 2, 884. 23181192 10.1038/srep00884 PMC3504926

[83] X. Li , Y. Jung , K. Sakimoto , T.‐H. Goh , M. A. Reed , A. D. Taylor , Energy Environ. Sci. 2013, 6, 887.

[84] X. Li , Y. Jung , J.‐S. Huang , T. Goh , A. D. Taylor , Adv. Energy Mater. 2014, 4, 1400186.

[85] F. Wang , D. Kozawa , Y. Miyauchi , K. Hiraoka , S. Mouri , Y. Ohno , K. Matsuda , Nat. Commun. 2015, 6, 6305. 25692264 10.1038/ncomms7305

[86] W. Xu , S. Wu , X. Li , M. Zou , L. Yang , Z. Zhang , J. Wei , S. Hu , Y. Li , A. Cao , Adv. Energy Mater. 2016, 6, 1600095.

[87] K. Cui , Y. Qian , Il Jeon , A. Anisimov , Y. Matsuo , E. I. Kauppinen , S. Maruyama , Adv. Energy Mater. 2017, 7, 1700449.

[88] H. Wu , X. Zhao , Y. Sun , L. Yang , M. Zou , H. Zhang , Y. Wu , L. Dai , Y. Shang , A. Cao , Sol. RRL 2019, 3, 1900147.

[89] Y. Qian , Il Jeon , Ya‐L Ho , C. Lee , S. Jeong , C. Delacou , S. Seo , A. Anisimov , E. I. Kaupinnen , Y. Matsuo , Y. Kang , H.‐S. Lee , D. Kim , J.‐J. Delaunay , S. Maruyama , Y. Matsuo , Adv. Energy Mater. 2020, 10, 1902389.

[90] D. D. Tune , N. Mallik , H. Fornasier , B. S. Flavel , Adv. Energy Mater. 2020, 10, 1903261.

[91] J. Chen , Y. Shen , J. Guo , B. Chen , J. Fan , F. Li , H. Liu , Y. Xu , Y. Mai , Appl. Phys. Lett. 2017, 110.

[92] L. Yang , J. Guo , J. Li , J. Yan , K. Ge , J. Jiang , H. Li , B. S. Flavel , B. Liu , J. Chen , J. Mater. Chem. C 2020, 8, 15684.

[93] J. Chen , Y. Shen , J. Guo , B. Chen , J. Fan , F. Li , B. Liu , H. Liu , Y. Xu , Y. Mai , Electrochim. Acta 2017, 247, 834.

[94] J. Chen , K. Ge , C. Zhang , J. Guo , L. Yang , D. Song , F. Li , Z. Xu , Y. Xu , Y. Mai , ACS Appl. Mater. Interfaces 2018, 10, 44896. 10.1021/acsami.8b17379 30499658

[95] J. Chen , L. Yang , K. Ge , B. Chen , Y. Shen , J. Guo , H. Liu , Y. Xu , J. Fan , Y. Mai , Appl. Phys. Lett. 2017, 111.

[96] W. Li , X. Wang , J. Guo , X. Zhang , B. Chen , J. Chen , Q. Gao , X. Yang , F. Li , J. Wang , D. Song , S. Wang , H. Li , J. Chen , J. Wang , Adv. Energy and Sustainability Res. 2023, 4, 2200154.

[97] J. Wang , M. Musameh , Y. Lin , J. Am. Chem. Soc. 2003, 125, 2409. 10.1021/ja028951v 12603125

[98] M. Casciola , G. Alberti , M. Sganappa , R. Narducci , J. Power Sources 2006, 162, 145.

[99] M.‐H. Lee , L. Chen , N. Li , F. Zhu , J. Mater. Chem. C 2017, 5, 10555.

[100] K. Ge , J. Chen , B. Chen , Y. Shen , J. Guo , F. Li , Z. Wang , J. Fan , H. Liu , Y. Xu , Physica Status Solidi (RRL)–Rapid Research Letters 2017, 11, 1700206.

[101] M. Lozac'h , S. Nunomura , H. Sai , K. Matsubara , Sol. Energy Mater. Sol. Cells 2018, 185, 8.

[102] S. Ardali , G. Atmaca , S. Lisesivdin , T. Malin , V. Mansurov , K. Zhuravlev , E. Tiras , Physica Status Solidi 2015, 252, 1965.

[103] H. Kim , C. M. Gilmore , A. Piqué , J. S. Horwitz , H. Mattoussi , H. Murata , Z. H. Kafafi , D. B. Chrisey , J. Appl. Phys. 1999, 86, 6461.

[104] M. J. Price , J. M. Foley , R. A. May , S. Maldonado , Appl. Phys. Lett. 2010, 97.

[105] J. Chen , Lu Wan , H. Li , J. Yan , J. Ma , B. Sun , F. Li , B. S. Flavel , Adv. Funct. Mater. 2020, 30, 2004476.

[106] J. Chen , D. D. Tune , K. Ge , H. Li , B. S. Flavel , Adv. Funct. Mater. 2020, 30, 2000484.

[107] M. Pfohl , K. Glaser , J. Ludwig , D. D. Tune , S. Dehm , C. Kayser , A. Colsmann , R. Krupke , B. S. Flavel , Adv. Energy Mater. 2016, 6, 1501345.

[108] M. Pfohl , K. Glaser , A. Graf , A. Mertens , D. D. Tune , T. Puerckhauer , A. Alam , Li Wei , Y. Chen , J. Zaumseil , A. Colsmann , R. Krupke , B. S. Flavel , Adv. Energy Mater. 2016, 6, 1600890.

[109] M. Zheng , Single‐Walled Carbon Nanotubes: Preparation, Properties and Applications 2019, 164.

[110] Q. Gao , J. Yan , H. Li , J. Chen , X. Yang , Y. Bai , X. Zhang , B. Chen , J. Guo , W. Duan , K. Han , F. Li , J. Wang , D. Song , S. Wang , B. S. Flavel , J. Chen , W. Duan , Carbon 2023, 202, 437.

[111] X. Yang , H. Xu , W. Liu , Q. Bi , L. Xu , J. Kang , M. N. Hedhili , B. Sun , X. Zhang , S. De Wolf , Adv. Electron. Mater. 2020, 6, 2000467.

[112] E. Kobayashi , S. De Wolf , J. Levrat , A. Descoeudres , M. Despeisse , F.‐J. Haug , C. Ballif , Sol. Energy Mater. Sol. Cells 2017, 173, 49.

[113] T. Urban , K. Krügel , J. Heitmann , Energy Procedia 2017, 124, 935.

[114] L. Wan , C. Zhang , K. Ge , X. Yang , F. Li , W. Yan , Z. Xu , L. Yang , Y. Xu , D. Song , J. Chen , Adv. Energy Mater. 2020, 10.

[115] J. Yan , C. Zhang , H. Li , X. Yang , Lu Wan , F. Li , K. Qiu , J. Guo , W. Duan , A. Lambertz , W. Lu , D. Song , K. Ding , B. S. Flavel , J. Chen , A. Lambertz , Adv. Sci. 2021, 8, 2102027. 10.1002/advs.202102027 PMC8529485 34473427

[116] J. Yan , C. Zhang , H. Li , X. Yang , L. Wan , F. Li , K. Qiu , J. Guo , W. Duan , A. Lambertz , W. Lu , D. Song , K. Ding , B. S. Flavel , J. Chen , Adv. Sci. 2021, 8. 10.1002/advs.202102027 PMC8529485 34473427

[117] Q. Gao , J. Yan , H. Li , J. Chen , X. Yang , Y. Bai , X. Zhang , B. Chen , J. Guo , W. Duan , K. Han , F. Li , J. Wang , D. Song , S. Wang , B. S. Flavel , J. Chen , Carbon 2023, 202, 437.

[118] S. Iijima , T. Wakabayashi , Y. Achiba , J. Phys. Chem. 1996, 100, 5843.

[119] G. Li , Y. Li , H. Liu , Y. Guo , Y. Li , D. Zhu , Chem. Commun. 2010, 46, 3258. 10.1039/b922733d 20442882

[120] A. S. A. Ahmed , W. Xiang , A. Gu , X. Hu , I. A. Saana , X. Zhao , New J. Chem. 2018, 42, 11723.

[121] M. Batmunkh , M. Bat‐Erdene , J. G. Shapter , Adv. Energy Mater. 2018, 8, 1701832.

[122] L. Yu , A. S. R. Bati , T. S. L. Grace , M. Batmunkh , J. G. Shapter , Adv. Energy Mater. 2019, 9, 1901063.

[123] Y. Li , L. Xu , H. Liu , Y. Li , Chem. Soc. Rev. 2014, 43, 2586. 10.1039/c3cs60388a 24445869

[124] C. Zhang , B. Chen , X. Zhang , J. Yan , J. Chen , Q. Gao , X. Zhou , J. Guo , F. Li , J. Wang , D. Song , S. Wang , J. Chen , Solar RRL 2022, 6.

[125] C. Battaglia , A. Cuevas , S. De Wolf , Energy Environ. Sci. 2016, 9, 1576.

[126] K. Yoshikawa , H. Kawasaki , W. Yoshida , T. Irie , K. Konishi , K. Nakano , T. Uto , D. Adachi , M. Kanematsu , H. Uzu , K. Yamamoto , Nat. Energy 2017, 2, 8.

[127] Y. Bai , Q. Gao , B. Chen , W. Li , X. Zhang , D. Yang , X. Yang , J. Yan , J. Chen , J. Wang , D. Song , S. Wang , H. Li , B. S. Flavel , J. Chen , J. Wang , Small Structures 2023, 2200375.

[128] E. Kobayashi , Y. Watabe , R. Hao , T. S. Ravi , Prog. Photovoltaics 2016, 24, 1303.

[129] B. S. Khan , A. Mukhtar , T. Mehmood , M. Tan , J. Nanosci. Nanotechnol. 2016, 16, 9900. 10.1166/jnn.2018.13970 29448577

[130] M. Pfohl , D. D. Tune , A. Graf , J. Zaumseil , R. Krupke , B. S. Flavel , ACS Omega 2017, 2, 1171. 10.1021/acsomega.6b00468 PMC5377271 28393134

[131] R. He , S. Ren , C. Chen , Z. Yi , Y. Luo , H. Lai , W. Wang , G. Zeng , X. Hao , Y. Wang , Energy Environ. Sci. 2021, 14, 5759.

[132] E. Köhnen , P. Wagner , F. Lang , A. Cruz , B. Li , M. Roß , M. Jošt , A. B. Morales‐Vilches , M. Topič , M. Stolterfoht , D. Neher , L. Korte , B. Rech , R. Schlatmann , B. Stannowski , S. Albrecht , Sol. RRL 2021, 5.

[133] W. Qarony , M. I. Hossain , V. Jovanov , A. Salleo , D. Knipp , Y. H. Tsang , ACS Appl. Mater. Interfaces 2020, 12, 15086. 10.1021/acsami.9b21985 32141283

[134] M. De Bastiani , A. S. Subbiah , M. Babics , E. Ugur , L. Xu , J. Liu , T. G. Allen , E. Aydin , S. De Wolf , Joule 2022, 6, 1445.

[135] L. L. Yan , C. Han , B. Shi , Y. Zhao , X. D. Zhang , Mat. Today Nano 2019, 7.

[136] Bo Chen , Z. Yu , K. Liu , X. Zheng , Ye Liu , J. Shi , D. Spronk , P. N. Rudd , Z. Holman , J. Huang , Joule 2019, 3, 190.

[137] Ji. Y Hyun , K. M. Yeom , S.‐W. Lee , S. Bae , D. Choi , H. Song , D. Kang , J.‐K. Hwang , W. Lee , S. Lee , Y. Kang , H.‐S. Lee , J. H. Noh , D. Kim , ACS Appl. Energy Mater. 2022, 5, 5456.

[138] F. Sahli , J. Werner , B. A. Kamino , M. Bräuninger , R. Monnard , B. Paviet‐Salomon , L. Barraud , L. Ding , J. J. Diaz Leon , D. Sacchetto , G. Cattaneo , M. Despeisse , M. Boccard , S. Nicolay , Q. Jeangros , B. Niesen , C. Ballif , D. Sacchetto , Nat. Mater. 2018, 17, 826. 10.1038/s41563-018-0115-4 29891887

[139] A. Al‐Ashouri , E. Köhnen , B. Li , A. Magomedov , H. Hempel , P. Caprioglio , J. A. Márquez , A. B. Morales Vilches , E. Kasparavicius , J. A. Smith , N. Phung , D. Menzel , M. Grischek , L. Kegelmann , D. Skroblin , C. Gollwitzer , T. Malinauskas , M. Jost , G. Matic , B. Rech , R. Schlatmann , M. Topic , L. Korte , A. Abate , B. Stannowski , D. Neher , M. Stolterfoht , T. Unold , V. Getautis , S. Albrecht , Science 2020, 370, 1309. 33303611 10.1126/science.abd4016

[140] K. A. Bush , A. F. Palmstrom , Z. J. Yu , M. Boccard , R. Cheacharoen , J. P. Mailoa , D. P. Mcmeekin , R. L. Z. Hoye , C. D. Bailie , T. Leijtens , I. M. Peters , M. C. Minichetti , N. Rolston , R. Prasanna , S. Sofia , D. Harwood , W. Ma , F. Moghadam , H. J. Snaith , T. Buonassisi , Z. C. Holman , S. F. Bent , M. D. Mcgehee , T. Leijtens , Nat. Energy 2017, 2, 7.

[141] F. Fu , J. Li , T. C.‐J. Yang , H. Liang , A. Faes , Q. Jeangros , C. Ballif , Yi Hou , Y. Hou , Adv. Mater. 2022, 34, 2106540. 10.1002/adma.202106540 35060205

[142] National Renewable Energy Laboratory , Best Research Cell Efficiency Chart, https://www.nrel.gov/pv/cell‐efficiency.html (December 2023).

[143] H. Hu , X. Zhou , J. Chen , D. Wang , D. Li , Y. Huang , L. Zhang , Y. Peng , F. Wang , J. Huang , Energy & Environmental Materials 2023, 6, e12322.

[144] J. Liu , M. De Bastiani , E. Aydin , G. T. Harrison , Y. Gao , R. R. Pradhan , M. K. Eswaran , M. Mandal , W. Yan , A. Seitkhan , M. Babics , A. S. Subbiah , E. Ugur , F. Xu , L. Xu , M. Wang , A. Ur Rehman , A. Razzaq , J. Kang , R. Azmi , A. A. Said , F. H. Isikgor , T. G. Allen , D. Andrienko , U. Schwingenschlögl , F. Laquai , S. De Wolf , Science 2022, 377, 306. 10.1126/science.abn8910 35737811

[145] A. S. Subbiah , F. H. Isikgor , C. T. Howells , M. De Bastiani , J. Liu , E. Aydin , F. Furlan , T. G. Allen , F. Xu , S. Zhumagali , S. Hoogland , E. H. Sargent , I. Mcculloch , S. De Wolf , ACS Energy Lett. 2020, 5, 3040.

[146] L. L. Yan , C. Han , B. Shi , Y. Zhao , X. D. Zhang , Materials Today Nano 2019, 7, 100045.

[147] E. Aydin , T. G. Allen , M. De Bastiani , L. Xu , J. Ávila , M. Salvador , E. Van Kerschaver , S. De Wolf , Nat. Energy 2020, 5, 859.

[148] T. Duong , H. Pham , T. C. Kho , P. Phang , K. C. Fong , Di Yan , Y. Yin , J. Peng , Md. A Mahmud , S. Gharibzadeh , B. A. Nejand , I. M. Hossain , M. R. Khan , N. Mozaffari , Y. Wu , H. Shen , J. Zheng , H. Mai , W. Liang , C. Samundsett , M. Stocks , K. Mcintosh , G. G. Andersson , U. Lemmer , B. S. Richards , U. W. Paetzold , A. Ho‐Ballie , Y. Liu , D. Macdonald , A. Blakers , et al., Adv. Energy Mater. 2020, 10, 1903553.

[149] L. Mao , T. Yang , H. Zhang , J. Shi , Y. Hu , P. Zeng , F. Li , J. Gong , X. Fang , Y. Sun , X. Liu , J. Du , A. Han , L. Zhang , W. Liu , F. Meng , X. Cui , Z. Liu , M. Liu , Y. Sun , Adv. Mater. 2022, 34, 2206193. 10.1002/adma.202206193 35985840

[150] F. Khan , B. D. Rezgui , M. T. Khan , F. Al‐Sulaiman , Renewable Sustainable Energy Rev. 2022, 165, 112553.

[151] Y. Li , B. Shi , Q. Xu , L. Yan , N. Ren , Y. Chen , W. Han , Q. Huang , Y. Zhao , X. Zhang , Adv. Energy Mater. 2021, 11, 2102046.

[152] A. Walter , B. A. Kamino , S.‐J. Moon , P. Wyss , J. J. D. Leon , C. Allebé , A. Descoeudres , S. Nicolay , C. Ballif , Q. Jeangros , Energy Advances 2023. 10.1039/d3ya00048f PMC10634458 38013933

[153] C. Messmer , B. S. Goraya , S. Nold , P. S. C. Schulze , V. Sittinger , J. Schön , J. C. Goldschmidt , M. Bivour , S. W. Glunz , M. Hermle , Prog. Photovoltaics 2021, 29, 759.

[154] S. Zhu , X. Yao , Q. Ren , C. Zheng , S. Li , Y. Tong , B. Shi , S. Guo , L. Fan , H. Ren , C. Wei , B. Li , Y. Ding , Q. Huang , Y. Li , Y. Zhao , X. Zhang , H. Ren , Nano Energy 2018, 45, 280.

[155] J. Yan , C. Zhang , H. Li , X. Yang , Lu Wan , F. Li , K. Qiu , J. Guo , W. Duan , A. Lambertz , W. Lu , D. Song , K. Ding , B. S. Flavel , J. Chen , Adv. Sci. (Weinh) 2021, 8, e2102027. 34473427 10.1002/advs.202102027 PMC8529485

[156] T. Dürkop , S. A. Getty , E. Cobas , M. S. Fuhrer , Nano Lett. 2004, 4, 39.

[157] L. Yang , P. Kim , H. M. Meyer , S. Agnihotri , J. Colloid Interface Sci. 2009, 338, 134. 10.1016/j.jcis.2009.06.017 19635621

[158] M. L. Jue , S. F. Buchsbaum , C. Chen , S. J. Park , E. R. Meshot , K. J. J. Wu , F. Fornasiero , Adv. Sci. 2020, 7, 2001670. 10.1002/advs.202001670 PMC7740080 33344119

[159] G. Chen , T. M. Paronyan , E. M. Pigos , A. R. Harutyunyan , Sci. Rep. 2012, 2, 343. 22461974 10.1038/srep00343 PMC3315270

[160] H. Shen , T. Hu , H. Huang , D. Wu , J. Xia , J. Mater. Sci.: Mater. Electron. 2022, 33, 12092.

[161] L. Yang , B. Chen , K. Ge , J. Guo , F. Li , L. Yang , Y. Xu , D. Song , X. Yang , J. Mater. Sci. 2023, 58, 3708.

[162] W. Li , X. Wang , J. Guo , X. Zhang , B. Chen , J. Chen , Q. Gao , X. Yang , F. Li , J. Wang , D. Song , S. Wang , H. Li , J. Chen , Advanced Energy and Sustainability Research 2022, 4.

[163] R. B Weisman , S. M. Bachilo , Nano Lett. 2003, 3, 1238.

[164] M. Gong , T. A. Shastry , Q. Cui , R. R. Kohlmeyer , K. A. Luck , A. Rowberg , T. J. Marks , M. F. Durstock , H. Zhao , M. C. Hersam , ACS Appl. Mater. Interfaces 2015, 7, 7435. 10.1021/acsami.5b01536 25797180

[165] D. J. Bindl , M.‐Y. Wu , F. C. Prehn , M. S. Arnold , Nano Lett. 2011, 11, 460. 10.1021/nl1031343 21166422

[166] C. M. Isborn , C. Tang , A. Martini , E. R. Johnson , A. Otero‐De‐La‐Roza , V. C. Tung , J. Phys. Chem. Lett. 2013, 4, 2918.

[167] R. M. Jain , R. Howden , K. Tvrdy , S. Shimizu , A. J. Hilmer , T. P. Mcnicholas , K. K. Gleason , M. S. Strano , Adv. Mater. 2012, 24, 4439. 10.1002/adma.201202088 22740144

[168] J. Wang , X. Jin , Z. Liu , G. Yu , Q. Ji , H. Wei , J. Zhang , Ke Zhang , D. Li , Zi Yuan , J. Li , P. Liu , Y. Wu , Y. Wei , J. Wang , Q. Li , L. Zhang , J. Kong , S. Fan , K. Jiang , Z. Yuan , Nat. Catal. 2018, 1, 331.

[169] S. K. Samanta , M. Fritsch , U. Scherf , W. Gomulya , S. Z. Bisri , M. A. Loi , Acc. Chem. Res. 2014, 47, 2456. 10.1021/ar500141j 25025887

[170] M. C. Gwinner , F. Jakubka , F. Gannott , H. Sirringhaus , J. Zaumseil , ACS Nano 2012, 6, 548. 10.1021/nn203874a 22142143

[171] S. Park , H. W. Lee , H. Wang , S. Selvarasah , M. R. Dokmeci , Y. J. Park , S. N. Cha , J. M. Kim , Z. Bao , ACS Nano 2012, 6, 2496. 10.1021/nn204875a 22352426

[172] K. E. Moore , M. Pfohl , F. Hennrich , V. S. K. Chakradhanula , C. Kuebel , M. M. Kappes , J. G. Shapter , R. Krupke , B. S. Flavel , ACS Nano 2014, 8, 6764. 10.1021/nn500756a 24896840

[173] H. Li , G. Gordeev , S. Wasserroth , V. S. K. Chakravadhanula , S. K. C. Neelakandhan , F. Hennrich , A. Jorio , S. Reich , R. Krupke , B. S. Flavel , Nat. Nanotechnol. 2017, 12, 1182. 10.1038/nnano.2017.207 28967894

[174] J. Noffsinger , M. L. Cohen , Phys. Rev. B 2011, 83, 165420.

[175] A. H. Brozena , J. Moskowitz , B. Shao , S. Deng , H. Liao , K. J. Gaskell , Y. Wang , J. Am. Chem. Soc. 2010, 132, 3938. 10.1021/ja910626u 20178323

[176] Il Jeon , J. Yoon , U. Kim , C. Lee , R. Xiang , A. Shawky , J. Xi , J. Byeon , H. Mo Lee , M. Choi , S. Maruyama , Y. Matsuo , M. Choi , Adv. Energy Mater. 2019, 9, 1901204.

[177] K. E. Moore , B. S. Flavel , A. V. Ellis , J. G. Shapter , Carbon 2011, 49, 2647.

[178] D. B. Mitzi , L. L. Kosbar , C. E. Murray , M. Copel , A. Afzali , Nature 2004, 428, 303. 15029191 10.1038/nature02389

[179] W. Deng , Y. Xiao , B. Lu , L. Zhang , Y. Xia , C. Zhu , X. Zhang , J. Guo , X. Zhang , J. Jie , Adv. Mater. 2021, 33, 2005915. 10.1002/adma.202005915 33336501

[180] Y.‐H Chang , S.‐R. Tseng , C.‐Y. Chen , H.‐F. Meng , En‐C Chen , S‐Fu Horng , C.‐S. Hsu , Org. Electron. 2009, 10, 746.

[181] X. Ding , J. Liu , T. A. L. Harris , AIChE J. 2016, 62, 2508.

[182] X. Dai , Y. Deng , C. H. Van Brackle , J. Huang , International Journal of Extreme Manufacturing 2019, 1, 022004.

[183] A. Moridi , S. M. Hassani‐Gangaraj , M. Guagliano , M. Dao , Surf. Eng. 2014, 30, 395.

[184] S. Chung , K. Cho , T. Lee , Adv. Sci. 2019, 6, 1801445. 10.1002/advs.201801445 PMC6425446 30937255

[185] B. Kim , M. L. Geier , M. C. Hersam , A. Dodabalapur , Sci. Rep. 2017, 7, 39627. 28145438 10.1038/srep39627 PMC5286420

[186] R. Tortorich , J.‐W. Choi , Nanomaterials 2013, 3, 468. 10.3390/nano3030453 PMC5304649 28348344

[187] M. J. Park , C. Wang , D. H. Seo , R. R. Gonzales , H. Matsuyama , H. K. Shon , J. Membr. Sci. 2021, 620, 118901.

[188] Y. Rong , Y. Ming , W. Ji , Da Li , A. Mei , Y. Hu , H. Han , J. Phys. Chem. Lett. 2018, 9, 2713. 10.1021/acs.jpclett.8b00912 29738259

